# Different Topologies of Hg(II)‐Bispidine 1D Coordination Polymers: Dynamic Behavior in Solvent Adsorption and Exchange Processes

**DOI:** 10.1002/chem.202200420

**Published:** 2022-03-29

**Authors:** Martina Lippi, Andrea Murelli, Patrizia Rossi, Paola Paoli, Massimo Cametti

**Affiliations:** ^1^ Department of Chemistry Materials and Chemical Engineering “Giulio Natta” Politecnico di Milano Via Luigi Mancinelli, 7 20133 Milano Italy; ^2^ Department of Industrial Engineering Università degli Studi di Firenze Via S. Marta 3 50136 Firenze Italy

**Keywords:** adsorption, coordination polymers, poly[*n*]catenanes, solvent exchange, X-ray structures

## Abstract

One‐dimensional (1D) coordination polymers (CPs) featuring three different topologies, comprising zig‐zag, ribbon‐like and poly‐[n]‐catenane structures, were obtained by reaction of Hg(II) ions with a novel bispidine ligand **L3**, and structurally characterized by SC‐ and P‐XRD methods. The CPs obtained in the form of microcrystalline powders were tested for their ability to undergo solvent adsorption and exchange by P‐XRD and ^1^H NMR spectroscopy. The extent of their dynamic behavior was then correlated to their structural features, highlighting the role of interchain interactions established among their constituting linear arrays. Zig‐zag CPs proved to be resilient to external chemical stimuli, while they differently respond to thermal treatments, depending on the solvent originally included within the CP. In the case of polycatenated structures, we observed transformations where the original topology was maintained upon guest exchange, but also cases where it changed to zig‐zag, even under solid/vapor conditions (i. e., no complete dissolution of the CP). Given the presence of linear interconnected 1D channels, **3** **⋅** **ClBz‐polycatenane^Pwd^** is also able to trap volatile guests such as *n*‐hexane when exposed to its vapors.

## Graphical abstract

**By reacting the** novel bispidine ligand **L3** with HgCl_2_, various coordination polymers having three different topologies (zig‐zag, ribbon‐like and poly[n]catenane), but all constituted by linear 1D arrays, can be formed. Their ability to undergo structural transformation via adsorption or solvent exchange has been reported and related to their structural features.

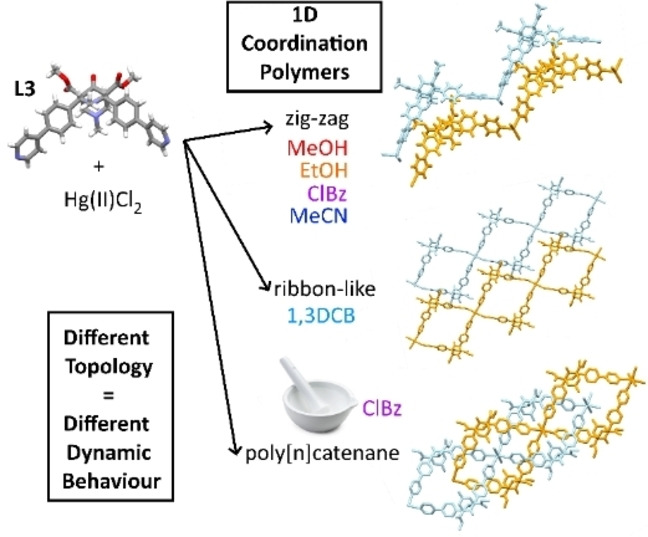

## Introduction

Over the last decades, we have witnessed an incredible advance in solid‐state and material chemistry which has been strongly kept up by new developments in the field of coordination polymers (CPs).^[1]^ Made upon the combination of organic ligands and metal salts, or clusters, CPs feature several useful properties, such as intriguing electronic, magnetic and optical features, and high porosity,^[2]^ together with the possibility to control, to an ever increasing degree, the design of the entire framework from the selection of the basic components. This allows to design and create materials with specific structures and attributes to suit emerging application needs. CP dimensionality, which defines how the metal‐ligand coordination bonds extend within the framework, is one of the key features in determining the overall material properties, especially for adsorption application. In these regards, metal–organic frameworks (MOFs),^[3]^ most often being 3D CPs, are considered to be the materials of the future. With large pores and/or channels, and open frameworks, which can be maintained after solvent evacuation, MOFs are intrinsically porous and usually very robust.^[4]^ Thus, it is quite straightforward to associate their accessible internal space to advantageous adsorption capabilities. At the same time, non‐porous materials are conventionally disregarded as adsorbents. Recently, it has been shown that a certain degree of permeability to liquid and vapor guests is also present in organic molecular solids devoid of accessible void space within their lattice.^[5]^ Also, seminal works by Takamizawa, Vittal, Noro and others,^[6]^ revealed that 1D CPs composed of linear chains are indeed quite dynamic systems and very efficient for guest replacement and selective adsorption processes. In these systems, the presence of weak inter‐chain interactions^[7]^ among robust linear metal–organic arrays may nonetheless lead to crystal flexibility which in turn translates into dynamic properties and marked adsorption capabilities.[^6^, ^7^]

**Scheme 1:**
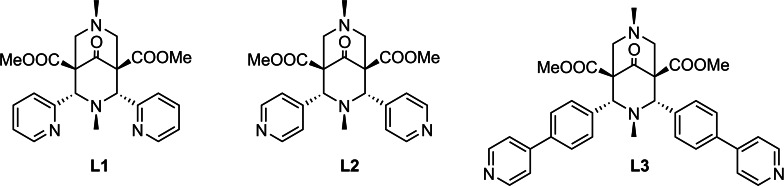
Chemical formulae for classic bispidine metal binder **L1**, and ligands **L2** and **L3** designed for coordinative polymerization.

In the course of our recent studies on 1D CPs with tunable adsorption properties,^[8]^ we have reported on the first bispidine based CPs, obtained by orienting the pyridine N donors of the classic bispidine metal binders,^[9]^ as in **L1**, in a divergent fashion, as in **L2** (Scheme 1). In combination with Mn(II) ions, mono and bi‐solvated CPs could be formed. They were characterized by ribbon‐like linear arrays made of ‐[Mn(Cl)_2_(**L2**)_2_]‐ units and proved to be capable to undergo various types of solvent exchange and adsorption reactions via solid/liquid or solid/vapor processes.

In this work, we have designed and synthesized a new ligand **L3**, decorated with extended lateral arms (Scheme 1), and we have investigated its ability to form novel and dynamic CPs when reacted with HgCl_2_ in the presence of different solvents (MeCN, MeOH, EtOH, toluene (MeBz), chlorobenzene (ClBz), 1,2‐dichlorobenzene (1,2‐DCB) and 1,3‐dichlorobenzene (1,3‐DCB) and under different reaction conditions (fast synthesis and slow crystallization from solution, solid‐state grinding, different T, etc…). Hg(II) was chosen on the basis of its adaptable coordination geometry, and its diamagnetism (a prerequisite for NMR analysis). We have isolated good quality single crystals of five different CPs with ligand **L3** (**3** **⋅** **MeOH^SC^**, **3** **⋅** **EtOH^SC^**, **3** **⋅** **ClBz^SC^**, **3** **⋅** **ClBz‐polycatenane^SC^** and **3** **⋅** **1,3‐DCB^SC^**), all characterized by linear 1D arrays, (containing MeOH, EtOH, ClBz and 1,3‐DCB as included solvents) but comprising three different topologies,^[10]^ such as zig‐zag, ribbon‐like and a more exotic poly‐catenane structure. We have also obtained several CPs in the form of microcrystalline powder phases characterized by P‐XRD and analyzed in terms of the amount of solvent present within the CP lattice by ^1^H NMR spectroscopy. We were able to reproduce all the above‐mentioned SC phases as microcrystalline powders (except for **3** **⋅** **1,3‐DCB^SC^**). These powder samples have been then tested for solvent exchange or adsorption in order to determine their dynamic behavior. As zig‐zag arrays do not show appreciable dynamic behavior, while polycatenated chains are subjected to drastic transformations (up to a topology change to zig‐zag), these data help to gather useful information which could lead to a correlation between CPs topology and solvent adsorption and/or exchange capability. In the case of polycatenated species, however, ribbon instability might have a role.

## SC‐XRD crystal structures

Single crystals of five different CPs made of ligand **L3** and HgCl_2_, suitable for X‐ray diffraction, were obtained through a slow crystallization process with a wide range of solvents by using the three‐layer crystallization method (Figure S5, Supporting Information).

**Scheme 2:**
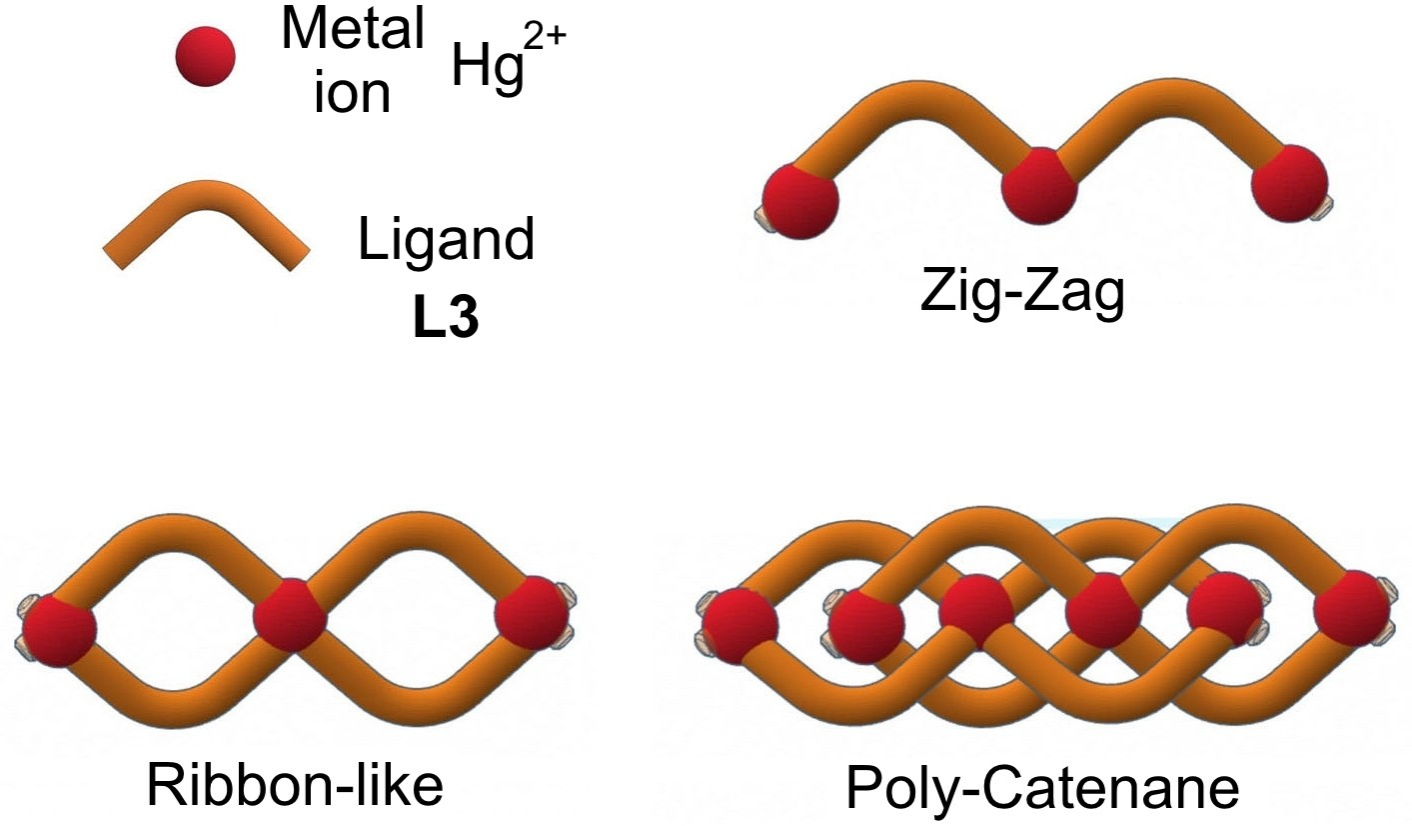
Cartoon of the different CP topologies encountered when **L3** is reacted with HgCl_2_ under various solvent content conditions (Supporting Information).

CPs belonging to the 1D family were formed in all cases. However, depending on the co‐crystallization solvent employed, they displayed three different topologies,^[10]^ comprising zig‐zag, ribbon‐like and poly‐catenane structures, as schematically represented in Scheme 2.

The mercury ion is tetra‐coordinated in **3** **⋅** **MeOH^SC^**, **3** **⋅** **EtOH^SC^** and **3** **⋅** **ClBz^SC^** while it exhibits a definitely less common hexa‐coordination in **3** **⋅** **ClBz‐polycatenane^SC^** and **3** **⋅** **1,3‐DCB^SC^**, as provided by the results of a search in the Cambridge Structural Database (CSD).^[11]^ In all five CPs, the chloride ions occupy two positions of the metal coordination sphere, being the remaining two, or four, coordination sites taken by the nitrogen atoms provided by the 4‐phenylpyridine groups of two, or four, different **L3** ligands. As to the first set of structures featuring a tetra‐coordinated mercury, Hg(II) metal can be described as in a distorted trigonal pyramidal environment with the apical position occupied by a nitrogen atom, as provided by the τ_4_ value (the four‐coordinated geometry index).^[12]^ In **3** **⋅** **1,3‐DCB^SC^** and **3** **⋅** **ClBz‐polycatenane^SC^** instead, the metal sits on a special position (an inversion center and a twofold axis, respectively) and adopts an octahedral coordination geometry with the four nitrogen atoms defining the equatorial plane. In all cases bond distances and angles (Table S3 in Supporting Information) are in the observed range for analogous Hg(II) complexes.^[11]^ However, in both octahedral complexes, the Hg−N distances are definitely long (2.55–2.65 Å for octahedral vs. 2.37–2.42 Å for tetracoordinated), most probably due to the mutual steric hindrance of the close 4‐phenylpyridine groups.

In **3 ⋅ MeOH^SC^**, **3 ⋅ EtOH^SC^** and **3 ⋅ ClBz^SC^** the mercury ion bridges two bispidine ligands originating a 1D zig‐zag motif, while in **3 ⋅ 1,3‐DCB^SC^** and **3 ⋅ ClBz‐polycatenane^SC^**, ribbon‐like arrays are formed, similarly to what occurred in the CPs obtained with L2 and previously reported.^[8]^ Notably, however, in 3 ⋅ ClBz‐polycatenane^SC^, two arrays are linked together by a mutual interpenetration which gives rise to a poly[n]catenane structure.

**Figure 1:**
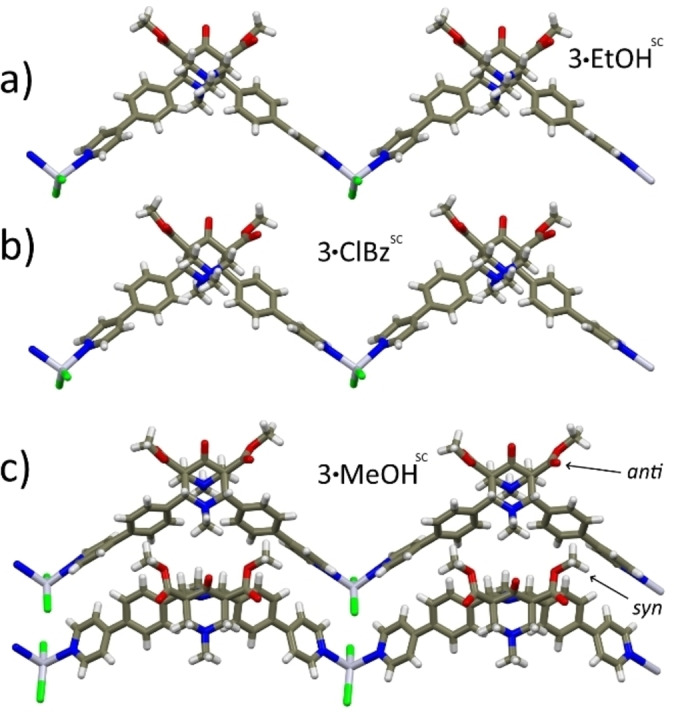
Views extending along the *c*‐axis direction of zig‐zag 1D‐CP arrays in a) **3** **⋅** **EtOH^SC^**, b) **3** **⋅** **ClBz** and c) **3** **⋅** **MeOH^SC^**. Color code: C=dark gray; H=white; N=blue; O=red; Cl=green; Hg=light violet.

Coordination polymers based on Hg(II) center are plenty,^[13]^ although considerably less common than those made with element of the same group, *viz*., Zn(II) or Cd(II); several examples exist with a tetracoordinated Hg(II) bound to two halides and two monodentate N donor ligands (similarly to the environment found in the SC structures described in Figure 1.[^13b^, ^13c^, ^13d^]

In all the crystal structures, **L3** adopts the usual chair‐chair conformation (Figure S6, Supporting Information), and it displays almost identical orientation of the pyridine moiety with respect to the phenyl rings, with angles between the two mean planes in the 31–43° range.^[14]^

CPs **3** **⋅** **MeOH^SC^**, **3** **⋅** **EtOH^SC^** and **3** **⋅** **ClBz^SC^** share a very similar crystal arrangement: all 1D CPs extend along the *c*‐axis direction in zig‐zag shaped chains (Figure 1). Moreover, **3** **⋅** **EtOH^SC^** and **3** **⋅** **ClBz^SC^** belong to the same space group (P2_1_/n) and have very similar unit‐cell dimensions (Table S1, Supporting Information), yet they differ for the relative orientation of the ester groups (*anti* and *syn*, respectively). The unit‐cell dimensions of **3** **⋅** **MeOH^SC^** are also quite similar, but the crystal symmetry is lower: space group P‐1 with two independent **L3**‐HgCl_2_ units, as a result of the different arrangement (*anti* and *syn*) adopted by the ester groups of the bispidine ligands, and generating all *anti*‐ and all *syn*‐type chains (Figure 1c). In all the zig‐zag structures, pockets defined by adjacent chains host solvent molecules (Figures S7, Supporting Information). Notably, the CPs pocket dimensions, evaluated by the distances separating the ester methyl groups and the centroids of the 4‐phenylpyridine moieties which overlook the cavity, appear correlated to the ligand ester groups conformation, rather than to the guest molecules volume: 8.9x8.3 Å and 8.8x8.1 Å for **3** **⋅** **MeOH^SC^** and **3** **⋅** **EtOH^SC^** *anti*‐chains, respectively; 10.9x7.5 Å and 11.9x7.4 Å for **3** **⋅** **MeOH^SC^** and **3** **⋅** **ClBz^SC^** *syn*‐chains, respectively. On this basis, we can rationalize the static disorder which affects the solvent molecules within the CP pockets. Bigger cavity volumes allow the solvent molecules to adopt different orientations, as well as different conformations.

**Figure 2:**
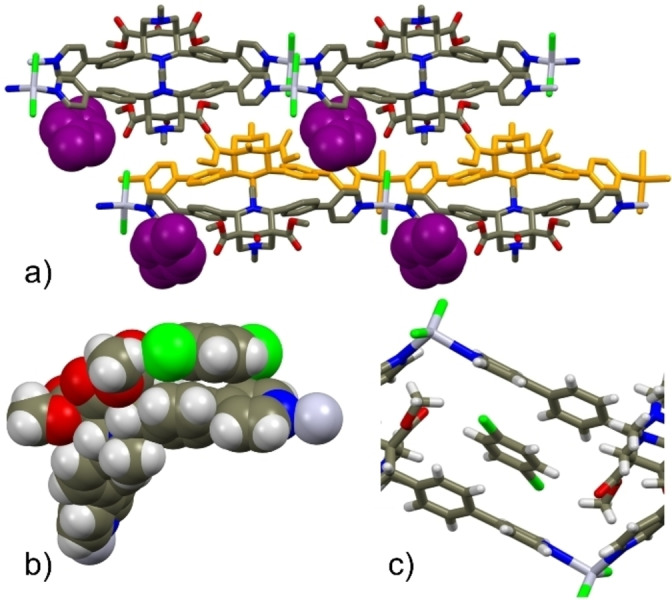
a) Crystal packing of **3** **⋅** **ClBz^SC^** viewed along the *b*‐axis direction (ClBz solvent is in CPK and purple, hydrogen atoms are omitted for clarity); b) specific π‐π interactions between a disordered ClBz solvent and the 4‐phenylpyridine moiety of **L3**; c) view of the intercalation of ClBz between two ligand units. Color code=dark gray; H=white; N=blue; O=red; Cl=green; Hg=light violet.

The packing of **3** **⋅** **ClBz^SC^**, representative of all zig‐zag structures, is shown in Figure 2a. In this case, two different orientations have been found for the chlorobenzene molecule (related by an inversion center) whose ring is found sandwiched between two sidearms of two different arrays (Figure 2b) and almost parallel to the mean plane defined by the 4‐phenylpyridine moieties (host‐guest π–π distance of ca. 3.7 Å) (Figure 2c). Finally, in **3** **⋅** **MeOH**, there are additional methanol molecules (occupancy factor 0.25) comprised between *anti*‐chains related by a translation along the *b* axis direction.

In all cases, solvent molecules are affected by disorder, thus solvent‐chains interactions cannot be reliably discussed. However, in silico removal of the solvents makes evident that they are hosted in isolated pockets corresponding to void of ca. 8.8 %, 6.8 % and 10.0 % of the total unit cell volume, for **3** **⋅** **MeOH^SC^**, **3** **⋅** **EtOH^SC^** and **3** **⋅** **ClBz^SC^**, respectively (Figure S8, Supporting Information).

Despite the overall structural similarities, different inter‐chain interactions hold together adjacent chains in the three CPs. In particular, in **3** **⋅** **MeOH^SC^**, symmetry related chains made of *anti* bispidine ligands are H‐bonded with one ester group oxygens and the central carbonyl as HB acceptors.^[15]^ On the contrary no significant inter‐array contacts involve the *syn‐*chains (Figure S9a, Supporting Information). The adjacent *anti*‐chains in **3** **⋅** **EtOH^SC^**, related by inversion centers, are bound through C=O⋅⋅⋅H bonds involving the oxygen atoms of one of the ester groups and the central carbonyl as HB acceptors (Figure S9b Supporting Information).^[16]^ Finally, in **3** **⋅** **ClBz^SC^** adjacent *syn*‐chains, related by an inversion center, are H‐bonded through the oxygen atom of an ester group and a Hg‐coordinated chloride ion (Figure S9c, Supporting Information).^[17]^ In all cases, Hirshfeld Surface analysis^[18]^ evidences that the above‐mentioned contacts are rather weak (Figure S10a‐d, Supporting Information). Nevertheless, the three zig‐zag structures are very efficiently packed as attested by the crystal density values which are quite alike in keeping with their high structural similarity: 1.665, 1.675 and 1.682 g/cm^3^, for **3** **⋅** **MeOH^SC^**, **3** **⋅** **EtOH^SC^** and **3** **⋅** **ClBz^SC^**, respectively. The lack of strong inter‐chain interactions, coupled with the high packing efficiency suggests that chains structural complementarity plays an important role in minimizing empty volume and, consequently, the potential energy of the system.

**Figure 3:**
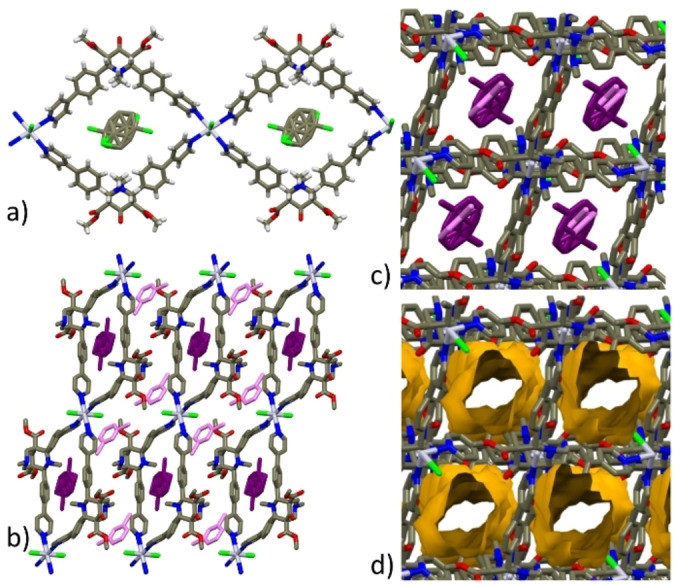
a) View of the ribbon‐like structure of **3** **⋅** **1,3‐DCB^SC^** extending along the *ac* bisector direction; b) crystal packing showing the two different guest solvent molecules (purple: hosted in the macrocyclic cavity; pink lodged between ribbons; c) packing view showing 1D channel filled with stacked 1,3‐DCB molecules and d) resulting voids by in‐silico removal of the solvent. Color code: C=dark gray; H=white; N=blue; O=red; Cl=green; Hg=light violet.

**Figure 4:**
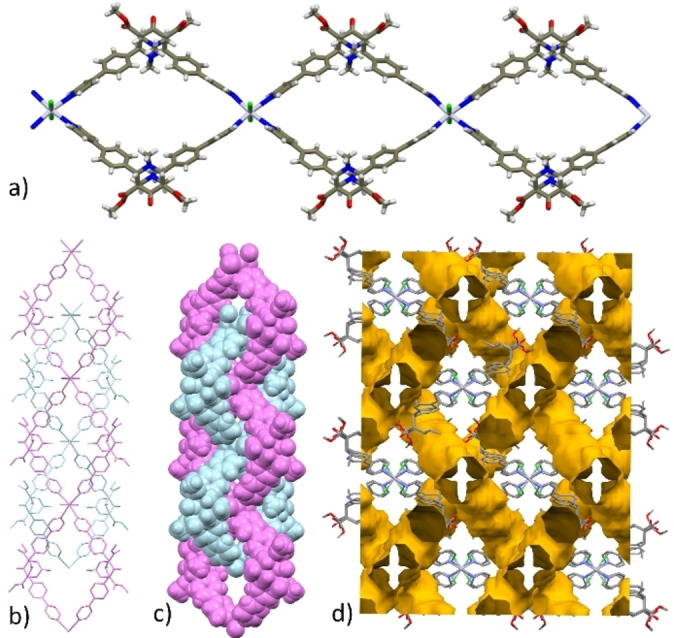
a) View of the ribbon‐like units in **3** **⋅** **ClBz‐polycatenane^SC^** extending along the *c*‐axis direction (color code: C=dark gray; H=white; N=blue; O=red; Cl=green; Hg=light violet; b) capped‐stick and c) CPK models of **3** **⋅** **ClBz‐polycatenane^SC^** showing two catenated ribbon‐like arrays (in cyan and purple); d) view of the packing along *c*‐axis with voids (ca. 25 %) generated by in‐silico removal of the solvent molecules. In b‐d) H atoms have been omitted for sake of clarity.

As previously noted, in **3** **⋅** **1,3‐DCB^SC^** and **3** **⋅** **ClBz‐polycatenane^SC^** ribbons are formed (Figures 3a and 4a), similarly to CPs previously reported with **L2**,^[8]^ extending along the *ac* bisector and the *c*‐axis directions, respectively. Examples of octahedral Hg(II) having two halides and four monodentate N based ligands are decidedly less common.^[19]^

The cavity defined by the macrocycles in the two CPs has comparable dimensions as estimated by the metal‐metal and opposing N−CH_3_ groups distances within the macrocycles (Hg‐ ‐ ‐Hg=19.8 Å; N−CH_3_‐ ‐ ‐N−CH_3_ =13.1, 12.3, 11.6 Å and Hg—Hg=19.4 Å; N−CH_3_‐ ‐ ‐N−CH_3_=14.5, 12.0, 11.7 Å, in **3** **⋅** **ClBz‐polycatenane^SC^** and **3** **⋅** **1,3‐DCB^SC^**, respectively), and it is decidedly larger than that obtained with **L2** having shorter sidearms (Hg‐ ‐ ‐Hg=11.8 Å; N−CH_3_‐ ‐ ‐N−CH_3_=11.8, 10.3, 7.9, 4.2 Å and Hg‐ ‐ ‐Hg=12.8 Å; N−CH_3_‐ ‐ ‐N−CH_3_=10.9, 6.7 and 6.5, 5.2 Å).[^8a^, ^20^] This makes interpenetration possible, and **3** **⋅** **ClBz‐polycatenane^SC^** indeed displays poly[n]catenation (see below).

In **3** **⋅** **1,3‐DCB^SC^**, the solvent molecules hosted in the macrocyclic cavity are coplanar with the HgN_4_ moieties (Figure 3a) but disordered over two orientations. Additional, not disordered 1,3‐dichlorobenzene molecules are lodged in cavities comprised between adjacent ribbons (Figure 3b) hold in place by a CH⋅⋅⋅O=C interactions (C6BH6B⋅⋅⋅O3, 2.414(4)Å, 172.9(4)°). Stacks of ordered and disordered solvent molecules are piled up along the *b*‐axis direction; while they alternate along the *bc* bisector direction lodged in linear channels (Figure 3c). The in silico removal frees ca. 27 % of space (Figure 3d). Weak inter‐ribbon interactions involve both carbonyl ester groups and the coordinated chloride ion.^[21]^

In the polycatenane structure **3** **⋅** **ClBz‐polycatenane^SC^**, the interlocked rings are almost perpendicular one to the other (angle between the mean planes defined by the HgN_2_ groups is about 77°) as evidenced in Figures 4b and c and are weakly bound through CH⋅⋅⋅Cl contacts (C10H10⋅⋅⋅Cl1, 2.855(1)Å, 131.8(3)°; C16H16⋅⋅⋅Cl1, 2.963(1)Å, 135.8(3)°). Weak inter‐polycatenane arrays interactions involve the hydrogen atoms of both methyl esters with the Hg‐coordinated chlorine and nitrogen atoms (C33H33B⋅⋅⋅Cl1, 2.722(1)Å, 141.8(4)°; C33H33A⋅⋅⋅N1, 2.621(4)Å, 137.0(3)°) and the oxygen atom provided by a different methyl ester group (C28H28C⋅⋅⋅O4, 2.592(3)Å, 134.4(3)°). Additional inter‐polycatenane chains contacts involve HB between the NCH_3_ group and one ester carbonyl oxygen (C35H35B⋅⋅⋅O2, 2.474(4)Å, 157.4(3)°). Poly[n]catenated CPs are quite rare, ^[22]^ despite considerable efforts in devising strategies for effective synthesis of mechanically interlocked systems. In this case, we would like to note that concatenation occurs between two linear polymeric units and not via link of single macrocycles, a more common situation; also, that we observed a crucial solvent effect,^[23]^ as replacing ClBz with 1,2‐ or 1,3‐DCB leads to completely different outcomes.

## Synthesis of microcrystalline powders

**Figure 5:**
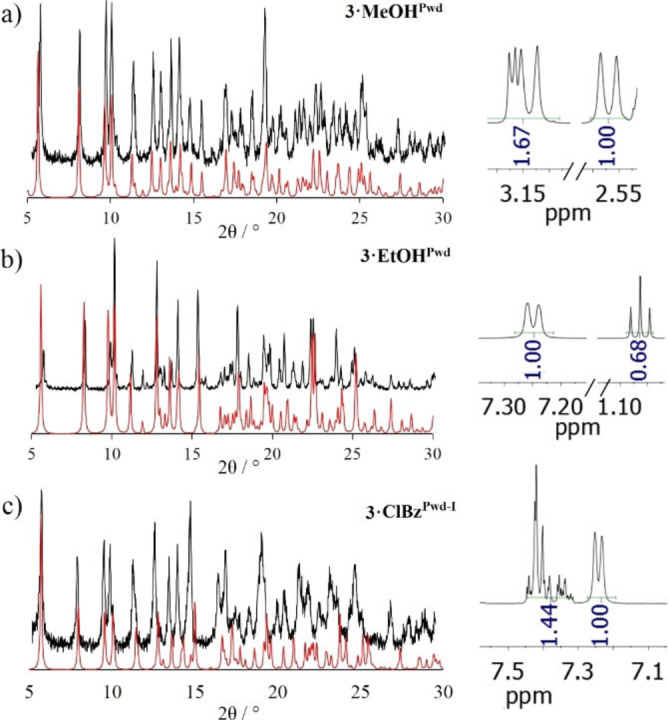
Experimental P‐XRD patterns (black) compared to the simulated data form SC (red) for a) **3** **⋅** **MeOH^Pwd^**; b) **3** **⋅** **EtOH^Pwd^** and c) **3** **⋅** **ClBz^Pwd−I^**. Each sample has been characterized also by ^1^H‐NMR (400 MHz, 302 K, DMSO‐*d6*), on the right. R values are=0.44, 0.46 and 0.58 for **3** **⋅** **MeOH^Pwd^**, **3** **⋅** **EtOH^Pwd^** and **3** **⋅** **ClBz^Pwd‐I^**, respectively (Table 1).

A fast crystallization procedure which can be described as mixing ligand **L3** dissolved in a given solvent with a MeOH solution of HgCl_2_ at r.t. generally leads to the precipitation of a white crystalline powder within few minutes (<30 mins) (Supporting Information). P‐XRD analysis of all products allows to phase‐match the fast crystallization products and the SC phases in the case of **3** **⋅** **MeOH^Pwd^**, **3** **⋅** **EtOH^Pwd^** and **3** **⋅** **ClBz^Pwd−I^** (see Figure 5).

**Figure 6:**
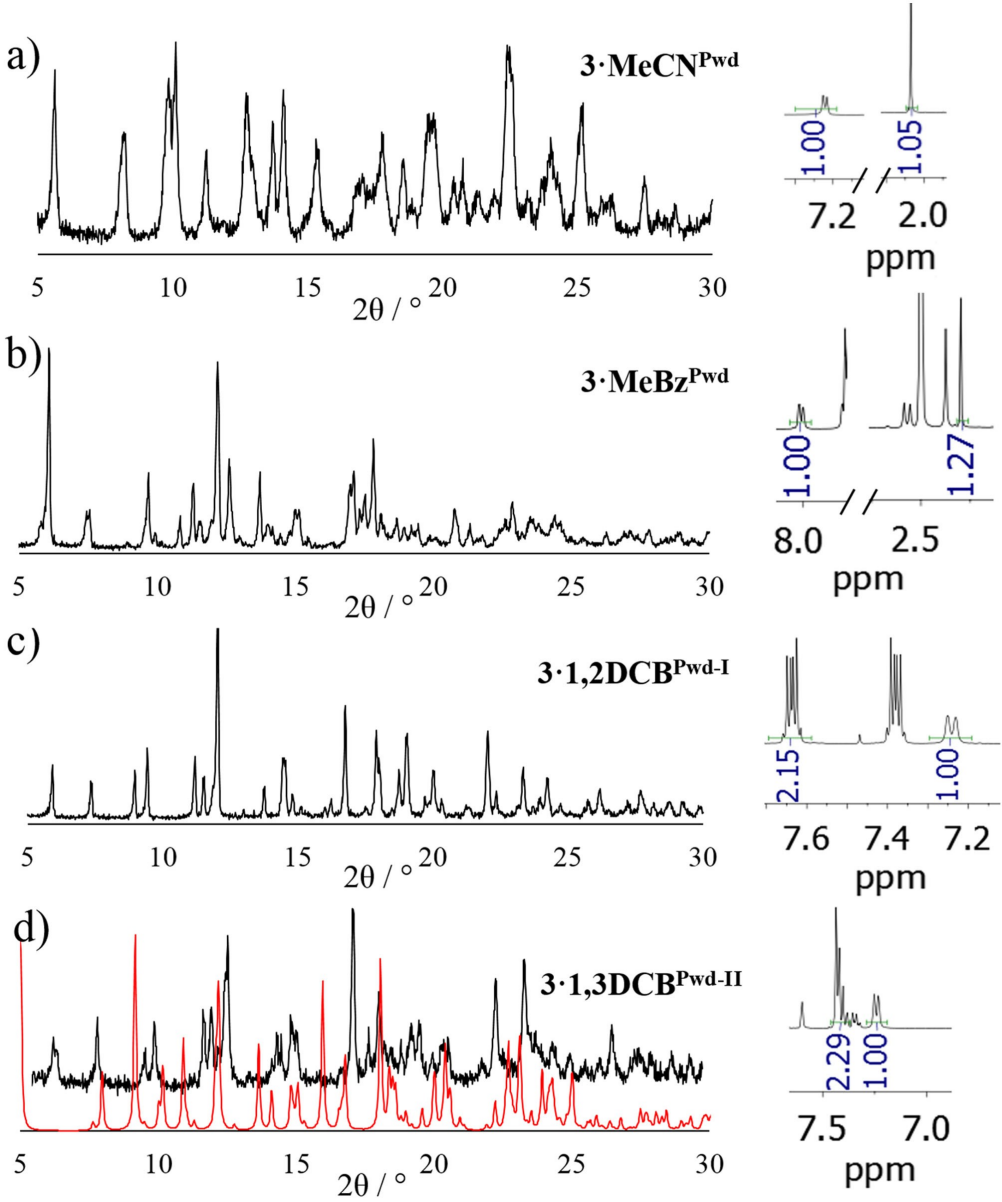
Experimental P‐XRD patterns (black) for a) **3** **⋅** **MeCN^Pwd^**; b) **3** **⋅** **MeBz^Pwd^** and c) **3** **⋅** **1,2DCB^Pwd−I^**; d) Experimental P‐XRD patterns (black) for **3** **⋅** **1,3DCB^Pwd−II^** compared to the simulated data form SC (red) of **3** **⋅** **1,3DCB^SC^**, showing no match; Each sample has been characterized also by ^1^H NMR (400 MHz, 302 K, DMSO‐*d6*), on the right. R values are 0.7, 0.85, 2.15 and 1.33 for **3** **⋅** **MeCN^Pwd^**, **3** **⋅** **MeBz^Pwd^**, **3** **⋅** **1,2DCB^Pwd−I^** and **3** **⋅** **1,3DCB^Pwd−II^**, respectively (Table 1).

In the case of fast synthesis with ClBz, a second phase, different than **3** **⋅** **ClBz^Pwd−I^** (Figure S11, Supporting Information) and named **3** **⋅** **ClBz^Pwd−II^** was obtained by keeping the fast crystallization mixture under stirring for a longer period of time (>2 h). We have also obtained other microcrystalline products, named **3** **⋅** **MeCN^Pwd^**, **3** **⋅** **MeBz^Pwd^**, **3** **⋅** **1,2DCB^Pwd−I^** and **3** **⋅** **1,3DCB^Pwd‐II^** which remain initially structurally unidentified due to the lack of the corresponding SC data (Figure 6). All attempts to reproduce the phase corresponding to ribbon‐like CP **3** **⋅** **1,3DCB^SC^** in microcrystalline form failed.

As to **3** **⋅** **MeCN^Pwd^**, a comparison between its P‐XRD pattern and those of **3** **⋅** **MeOH^Pwd^**, **3** **⋅** **EtOH^Pwd^** and **3** **⋅** **ClBz^Pwd−I^** allows to confidently establish that **3** **⋅** **MeCN^Pwd^** belongs to the zig‐zag family of CPs (see Figure S12, Supporting Information).

^1^H NMR analyses were carried out to quantify the amount of solvent trapped within the CPs for each microcrystalline phase after digesting it in DMSO‐*d_6_*.^[24]^ For each P‐XRD data set, a portion of the corresponding ^1^H NMR spectrum is shown (Figures 5 and 6). Solvent content has been expressed by the R factor which represents the number of solvent molecules per ligand, as determined by ^1^H NMR signal integral analysis. R values were compared with solvent content data extracted from SC‐XRD analyses and found in very good agreement, except for **3** **⋅** **MeOH^Pwd^** whose ^1^H NMR analysis provides a lower solvent content (R=ca. 0.5 to be compared with a R=1.25 from SC data).

**Figure 7:**
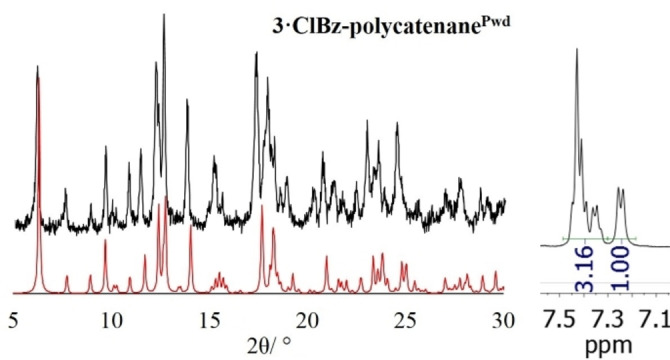
Experimental P‐XRD patterns of **3** **⋅** **ClBz‐polycatenane^Pwd^** (black) compared to the simulated data form SC of **3** **⋅** **ClBz‐polycatenane^SC^** (red). R value is 1.27 to be compared with 1.5 molecule of ClBz from SC‐XRD data (Table 1).

As to the polycatenated CP, although fast crystallization trials were not successful, we succeeded in obtaining a microcrystalline phase with a P‐XRD pattern matching with that of **3** **⋅** **ClBz‐polycatenane^SC^** by mechano‐synthesis. Indeed, by hand grinding ligand **L3** and 0.5 equivalents of HgCl_2_ in a mortar after addition of few drops of ClBz, CP **3** **⋅** **ClBz‐polycatenane^Pwd^** was obtained (see matching P‐XRD with simulated pattern of **3** **⋅** **ClBz‐polycatenane^SC^** in Figure 7). ^1^H NMR and SC‐XRD data on the ClBz content are also in very good agreement (compare 1.27 to 1.5 from Table 1).

**Table 1:** Summary of the SC and microcrystalline powder materials obtained in this work, including information on topology, M : L ratio and solvent content.

**Single Crystals** (composition)	**Microcrystalline Powders** Instant Synthesis=IS; Mechanochemistry=M; Thermal treatment=T; Exchange=Ex	**Match** between SC and P‐XRD data	**Topology (M/L ratio)**	**Solvent content** for samples obtained via instant synthesis^b^ (SC/NMR)	**Dynamic behavior** Thermal treatment=T Exchange=Ex Adsorption=Ads
**3⋅MeOH^SC^** [**L3 ⋅** HgCl_2_]⋅1.25MeOH	**3 ⋅ MeOH^Pwd^** (IS/Ex)	Y	zig‐zag (1 : 1)	(1.25/0.44)	T
**3 ⋅ EtOH^SC^** [**L3 ⋅** HgCl_2_]⋅0.5EtOH	**3 ⋅ EtOH^Pwd^** (IS)	Y	zig‐zag (1 : 1)	(0.5/0.46)	–
**3 ⋅ ClBz^SC^** [**L3 ⋅** HgCl_2_]⋅0.5ClBz	**3 ⋅ ClBz^Pwd‐I^** (IS/Ex)	Y	zig‐zag (1 : 1)	(0.5/0.58)	T
	**3 ⋅ ClBz^Pwd‐II^** (IS)	N	–	–	–
	**3 ⋅ MeCN^Pwd^** (IS/Ex)	–	zig‐zag^a^	(−/0.7)	T
	**3 ⋅ MeBz^Pwd^** (IS /Ex)	–	–	(−/0.85)	–
	**3 ⋅ 1,2DCB^Pwd‐I^** (IS)	–	–	(−/2.15)	–
	**3 ⋅ 1,2DCB^Pwd‐II^** (Ex)	–	–	–	Ex
	**3 ⋅ desolv^Pwd‐I^** (T)	–	zig‐zag ^a^	–	Ads
	**3 ⋅ desolv^Pwd‐II^** (T)	–	zig‐zag ^a^	–	Ads
**3 ⋅ 1,3‐DCB^SC^** [**L3**⋅Hg_0.5_Cl]⋅1.5(1,3‐DCB)	–	–	ribbon‐like (2 : 1)	(1.5/−)	–
	**3 ⋅ 1,3DCB^Pwd‐II^** (IS)	N	–	(−/1.33)	–
**3 ⋅ ClBz‐polycatenane^SC^** [**L3**⋅Hg_0.5_Cl]⋅1.5ClBz	**3 ⋅ ClBz‐polycatenane^Pwd^** M	Y	polycatenane (2 : 1)	(1.5/1.27)	Ex
	**3‐hexane^Pwd^** Ex	–	–	(−/0.82)	–

a) As determined by comparison among P‐XRD data; b) as determined by SC‐XRD and ^1^H NMR data.

In summary, we have obtained nine different CPs in the form of microcrystalline powders (Table 1).

## Dynamic behavior

The adsorption and dynamic properties of microcrystalline **3** **⋅** **MeCN^Pwd^**, **3** **⋅** **ClBz^Pwd−I^** and **3** **⋅** **MeOH^Pwd^** were tested by solid/liquid and solid/vapor adsorption experiments (Supporting Information).^[8]^ Each sample, initially containing one given solvent among MeOH, ClBz and MeCN, was exposed to vapors of, or dipped into, the other two guests and the resulting powder material was analyzed by P‐XRD. No changes were observed in all cases. Their similar behavior in terms of guest exchange aptitude reflects their crystal structure similarities: all belong to the *zig‐zag* family and the different type of inter‐chain interactions does not appear to affect the adsorption behavior. In fact, the high packing efficiency which characterizes the three zig‐zag CPs (in the 1.665–1.682 g/cm^3^ range) suggests a high crystal stability which can help to rationalize their inability to undergo any solvent induced transformations. An useful comparison can be made with the highly dynamic linear ribbon‐like CPs composed of ‐[Mn(Cl)_2_(**L2**)_2_]‐ repeating units, with nitrobenzene or chloroform trapped within.^[8]^ They are characterized as well by weak inter‐ribbon interactions (as suggested by Hirshfeld Surface analysis),^[18]^ but show a markedly less effective packing (d=1.327 and 1.457 g/cm^3^, respectively).

**Figure 8:**
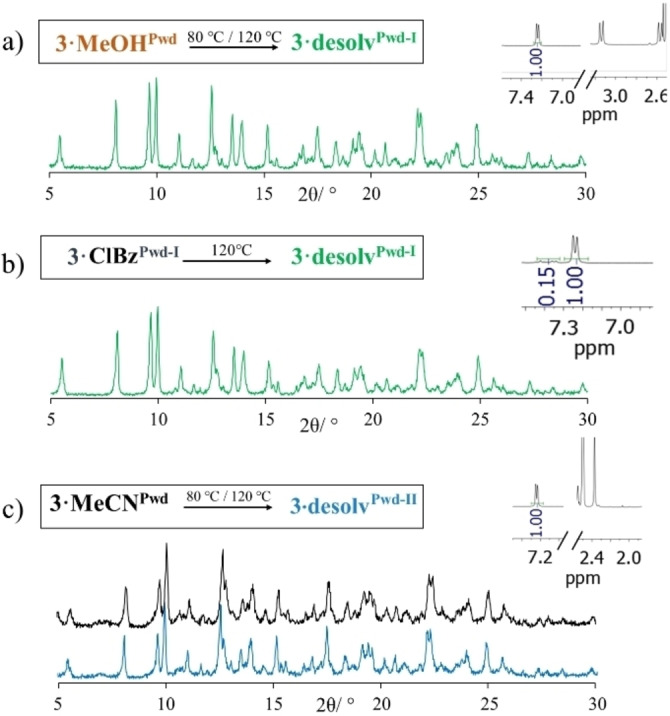
Summary of the thermal treatment performed on CPs and their outcome. Experimental P‐XRD pattern of CP obtained from thermal treatment of a) **3** **⋅** **MeOH^Pwd^** at 80 °C or 120 °C; b) **3** **⋅** **ClBz^Pwd−I^** at 120 °C and c) **3** **⋅** **MeCN^Pwd^** at 80 or 120 °C. Portions of relevant corresponding ^1^H NMR spectra are shown, indicating no solvent present (400 MHz, 302 K, DMSO‐*d6*).

Interestingly, thermal treatment of the three materials produces different outcomes. **3** **⋅** **MeOH^Pwd^** undergoes a phase transformation after treatment at 80 °C for 18 h to form a desolvated phase, a feature also corroborated by ^1^H NMR analysis (Figure S13a, Supporting Information), named **3** **⋅** **desolv^Pwd−I^** (its P‐XRD pattern is shown in Figure 8a). Same treatment generates no changes in the P‐XRD pattern of both **3** **⋅** **ClBz^Pwd−I^** and **3** **⋅** **MeCN^Pwd^**. By increasing T to 120 °C, **3** **⋅** **ClBz^Pwd‐I^** CP transforms into the desolvated phase **3** **⋅** **desolv^Pwd−I^** (Figure 8b), as seen by P‐XRD change and ^1^H NMR data on the solvent content (Figure S13b, Supporting Information). Notably, by looking at the P‐XRD pattern, the phase originating from **3** **⋅** **MeCN^Pwd^** still remains unperturbed at 120 °C (Figure 8c). However, the ^1^H NMR analysis of the sample shows no MeCN within the CP (Figure S13c, Supporting Information), and we named it **3** **⋅** **desolv^Pwd−II^**. As we do not have any SC data as structural reference for this phase, we can only note its marked similarities with **3** **⋅** **desolv^Pwd‐I^** and that it can undergo an isomorphous desolvation process,^[25]^ probably due to the different geometric (small and linear) and electronic (no HB donor capability) features of MeCN compared to MeOH and ClBz. In any case, its structure can sustain both the thermal treatment and the related loss of solvent, indicating structural resilience.

These findings highlight a different intrinsic stability of the three CPs which depends on the solvent content and thus on solvent‐arrays interactions, and not on the intrinsic different boiling points of the three species. Furthermore, when any of the above mentioned desolvated phases, regardless of the starting CP used to obtain them, is exposed to MeCN for 14 days, adsorption occurs leading to **3** **⋅** **MeCN^Pwd^** (confirmed by ^1^H NMR). This re‐adsorption process does not take place with MeOH or ClBz, but only with MeCN, demonstrating again the strong dependence of these processes to the solvent identity.

**Figure 9:**
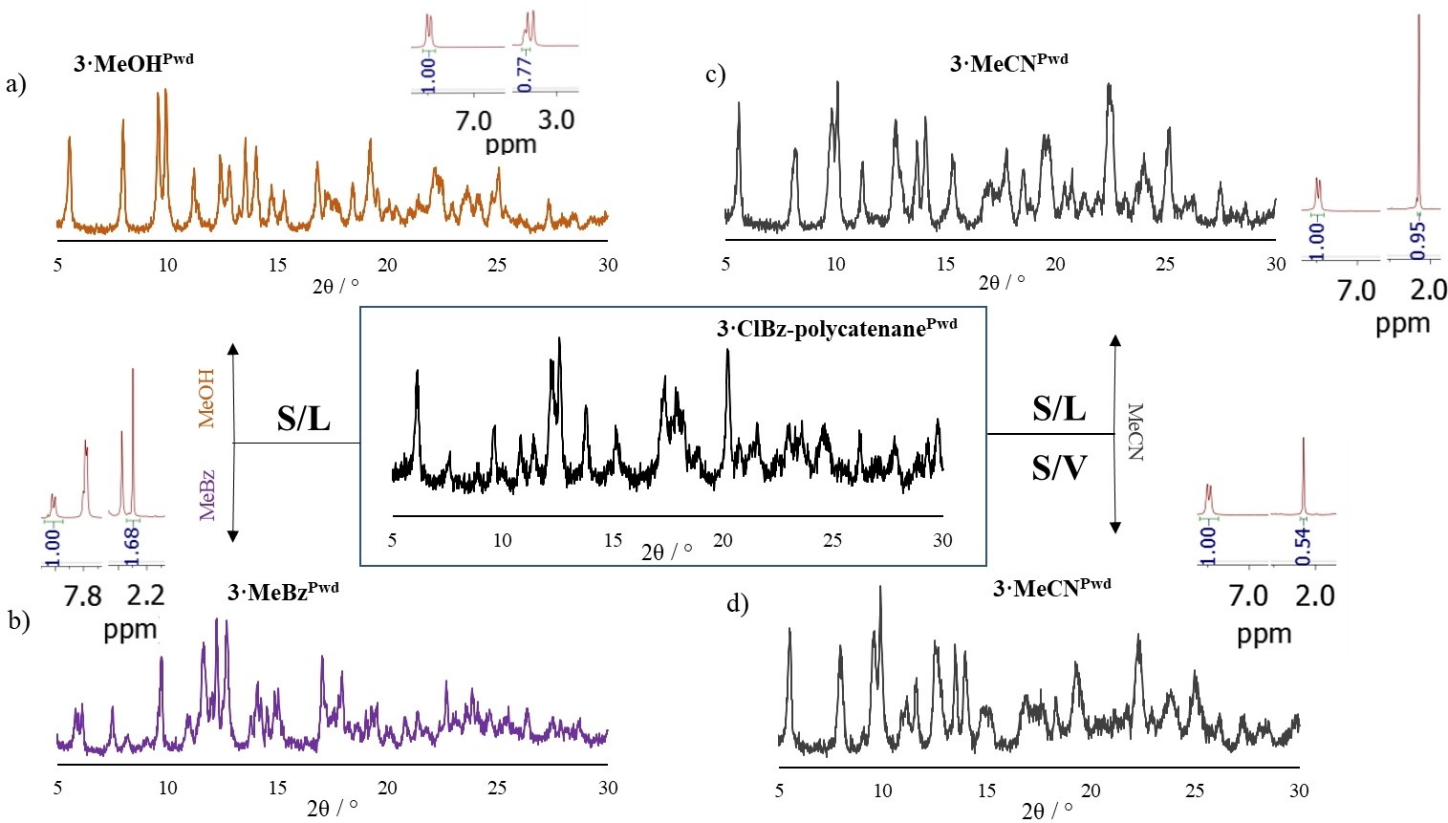
Summary of the guest‐induced transformations relative to CP **3** **⋅** **ClBz‐polycatenane^Pwd^**. Solid/liquid processes involving exposure to a) MeOH; b) MeCN, and c) MeBz leading to a CP of zig‐zag topology, structurally identical to the CPs obtained by instant synthesis carried out with those solvents (**3** **⋅** **MeOH^Pwd^**, **3** **⋅** **MeBz^Pwd−I^** and **3** **⋅** **MeCN^Pwd^**); d) solid/vapor process with MeCN leading to the same outcome of the analogous solid/liquid process. All phases were also characterized in terms of solvent content by ^1^H‐NMR analysis (relevant portions of corresponding spectra are shown). R values are a) 0.51, b) 0.63, c) 1.12 and d) 0.36.

Another interesting set of transformations pertains to **3** **⋅** **ClBz‐polycatenane^Pwd^** (Figure 9). Adsorption experiments by solid/liquid processes using MeOH (Figure 9a), MeBz (Figure 9b) and MeCN (Figure 9c) produced crystalline phases corresponding to the CPs obtained by instant synthesis with those solvents. In other words, the polycatenane ribbons open and convert into the zig‐zag like structures. This type of processes where the coordination bonds which make up the whole structures are necessarily broken, and topology changes, usually entails an at least partial dissolution of the CP. However, the analogous solid/vapor process also leads to the same changes, although only with MeCN (and not MeOH) (Figure 9d).

Solid/liquid processes using 1,2‐DCB lead to a new phase, named **3** **⋅** **1,2DCB^Pwd−II^** as its P‐XRD pattern is different than that of **3** **⋅** **1,2DCB^Pwd−I^** (obtained by instant synthesis), which nonetheless transforms to **3** **⋅** **ClBz^Pwd−I^** when dipped into ClBz (Figure S14 in Supporting Information). ^1^H NMR R values were found in line with those of the corresponding materials obtained by instant synthesis; except for the CP re‐obtained by solid/vapor process which shows a lower MeCN content (Figure 9d).

**Figure 10:**
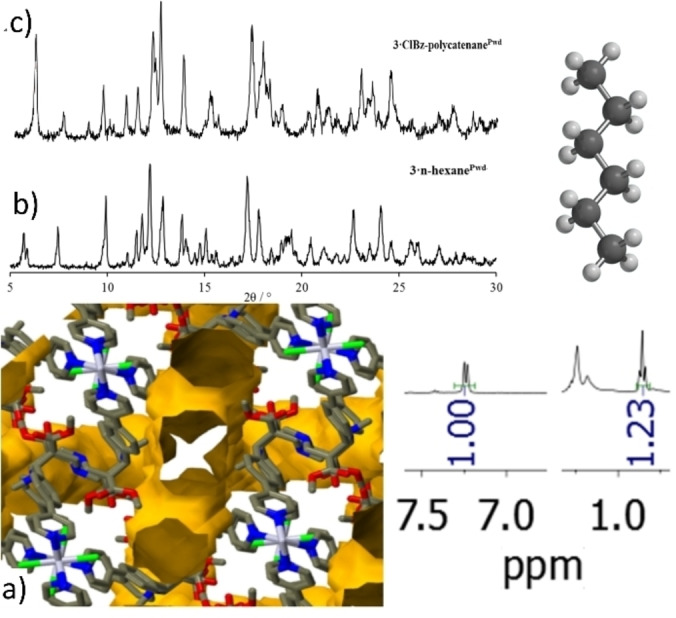
a) View of the void space (25 %) potentially available by removal of ClBz molecules from the interconnected channel of **3** **⋅** **ClBz‐polycatenane^SC^**. b) P‐XRD pattern of the CP obtained by exposure **of 3** **⋅** **ClBz‐polycatenane^Pwd^** (whose P‐XRD pattern is shown in c) to *n*‐hexane vapours.^1^H‐NMR R value is 0.82.

As said before, **3** **⋅** **ClBz‐polycatenane^Pwd^** possesses linear, highly interconnected channels with a section of approx. 4.5 Å (see Figures 4d and S15) which mainly develop parallel to the CP arrays. Initially, these pores are filled with ClBz molecules arranged head‐to‐tail into columns adorned with additional partially T‐stacked others (disordered) (Figure S15, Supporting Information). Thus, evacuation of these channels could lead to a potentially novel adsorbent material exploiting the available inter‐chain space. Thermal treatment at 80 °C and 120 °C sensibly reduces ClBz guest content, along with the crystallinity of the material and ^1^H NMR analysis also indicates some sort of degradation of the material (Figures S16 and S17, Supporting Information).^[26]^ This notwithstanding, solvent exchange processes at r.t. were attempted with a volatile linear guest: *n*‐hexane. By exposure of **3** **⋅** **ClBz‐polycatenane^Pwd^** to *n‐*hexane vapors for 1 month, or by dipping powders of polycatenated CP into *n‐*hexane for 3 days, we generated a novel phase (Figure 10b) and named it as **3‐hexane^Pwd^** for it contains hexane as demonstrated by ^1^H NMR analysis (R=0.82). Considering the channel volume of ca. 2000 Å^3^, the volume of hexane of ca. 130 Å^3^ and eight ligands per unit cell, the ^1^H NMR R value reported of 0.82 is in very good agreement with what could have been expected by applying Rebek's 0.55 rule (2000*0.55/130/8=1.06).^[27]^

## Conclusion

In this work, we have employed bispidine‐based ligand **L3** designed with extended side‐arms to produce, in combination with HgCl_2_, novel 1D CPs with different topologies. Common zig‐zag and ribbon‐like CPs, but also more exotic polycatenated species were obtained as SCs and/or microcrystalline powders, depending on the crystallization conditions, and characterized by SC‐XRD, P‐XRD and ^1^H NMR spectroscopy (Table 1).

Zig‐zag CPs such as **3** **⋅** **MeCN^Pwd^**, **3** **⋅** **ClBz^Pwd−I^** and **3** **⋅** **MeOH^Pwd^** proved to be resilient to external chemical stimuli, while they differently respond to thermal treatments. Highly dense packing due to a very efficient structural complementarity of the zig‐zag chains is probably responsible for their observed stability under exchange test conditions. They can also lead to desolvated species **3** **⋅** **desolv^Pwd−I^** and **3** **⋅** **desolv^Pwd−II^**, which maintain crystallinity up to 120 °C and, interestingly, they are able to selectively re‐adsorb only MeCN, and not MeOH or ClBz (Table 1).

Poly[n]catenated **3** **⋅** **ClBz‐polycatenane^Pwd^**, only obtained by a mechano‐chemical approach, featuring a quite uncommon structure characterized by two macrocyclic chains polycatenated into linear arrays, shows instead a more drastic attitude to respond to chemical stimuli, as it undergoes different transformations, also comprising a change of topology (to zig‐zag), in some cases, even by simple exposure to solvent vapors (e. g., to MeCN). This CP is also capable to adsorb *n*‐hexane into its linear interconnected channels by substitution of the initially included ClBz (Table 1).

These different dynamic behaviors, if compared to previous works related to analogous CPs constituted of ribbon‐like linear arrays (by using **L2** and MnCl_2_),^[8]^ highlight the direct relation between topology and dynamic behavior in terms of solvent guest adsorption and exchanges processes. Acquiring this knowledge is essential for the design of novel adsorbent materials with tailored properties, complementary perhaps to those of MOFs, which could meet future emerging needs in adsorption and separation technologies.

## Experimental Section

**Synthesis of L3**: 4‐(pyridin‐4‐yl)benzaldehyde (2 Eq, 4 g, 21.8 mmol) was added to a solution of dimethyl 1,3‐acetonidicarboxylate (1 Eq, 1.9 g, 10.9 mmol) dissolved in 15 mL of EtOH under stirring in a flask maintained at 0° with an ice bath. After 15 min, a solution of methylamine (1.5 Eq, 0.5 g, 16.7 mmol) in EtOH (2 mL) was added dropwise. The mixture was warmed to 40 °C and stirred for other 30 min, whereupon it was left to cool down to room temperature. The formation of a precipitate is observed. Then, a solution of methylamine (1.5 Eq, 0.5 g, 16.7 mmol) and formaldehyde (3.5 Eq, 1.15 g, 38.2 mmol) in 2 mL of EtOH was added to the suspension. The mixture was heated up to 55 °C for 1.5 h and finally cooled down to r.t. The white precipitate obtained was filtered and washed with cold EtOH. The mother liquors left were reduced to ca. 50 % in volume, and additional precipitate was filtered and washed with EtOH. The combined obtained solid (3.31 g) corresponded to a yield of ca. 50 %. ^1^H NMR (400 MHz, CDCl_3_) δ 8.72 ‐ 8.63 (m, 4H), 8.32 (dd, J=8.0, 1.8 Hz, 2H), 7.77 (dd, J=8.2, 2.0 Hz, 2H), 7.56 (dd, J=8.0, 2.0 Hz, 2H), 7.54 ‐ 7.50 (m, 4H), 7.25 (d, J=8.9 Hz, 2H), 4.59 (s, 2H), 3.77 (s, 6H), 3.30 ‐ 3.15 (m, 2H), 2.68 (d, J=12.1 Hz, 2H), 2.41 (s, 3H), 1.94 (s, 3H); ^13^C NMR (101 MHz, CDCl_3_) δ 203.9, 168.2 (2 C), 150.3 (4 C), 147.8 (2 C), 140.1 (2 C), 138.0 (2 C), 130.0 (2 C), 129.5 (2 C), 127.3 (2 C), 126.9 (2 C), 121.5 (4 C), 72.4 (2 C), 63.3 (2 C), 60.0 (2 C), 52.4 (2 C), 44.5, 43.2. ESI‐MASS: calcd. for C_35_H_35_N_4_O_5_=591.25 corresponding to [M+H)]^+^, found 591.4 m/z; and 609.4 m/z and 623.4 m/z corresponding to solvated [M+H+H_2_O]^+^ and [M+H+MeOH]^+^(Supporting Information). Elem. anal.: C, 71.17; H, 5.80; N, 9.49, calcd for C35H34 N4O5, found: C, 69.57; H, 5.98; N, 9.12.

Deposition Numbers 2150472 (for **3** **⋅** **EtOH** ^**SC**^), 2150473 (for **3** **⋅** **1,3‐DCB** ^**SC**^), 2150474 (for **3** **⋅** **ClBz** ^**SC**^), 2150475 (for **3** **⋅** **ClBz‐polycatenane** ^**SC**^), 2150476 (for **3** **⋅** **MeOH** ^**SC**^) contain the supplementary crystallographic data for this paper. These data are provided free of charge by the joint Cambridge Crystallographic Data Centre and Fachinformationszentrum Karlsruhe Access Structures service.

## Conflict of interest

The authors declare no conflict of interest.

1

## Supporting information

As a service to our authors and readers, this journal provides supporting information supplied by the authors. Such materials are peer reviewed and may be re‐organized for online delivery, but are not copy‐edited or typeset. Technical support issues arising from supporting information (other than missing files) should be addressed to the authors.

Supporting Information

## Data Availability

The data that support the findings of this study are available from the corresponding author upon reasonable request.
