# Supplementary material for: Different Topologies of Hg(II)‐Bispidine 1D Coordination Polymers: Dynamic Behavior in Solvent Adsorption and Exchange Processes
Source: Chemistry. 2022 Mar 29;28(26):e202200420. doi: 10.1002/chem.202200420 (PMC9311696; doi:10.1002/chem.202200420)
Supplement: Supplementary file 1 — Supporting Information [file CHEM-28-0-s001.pdf]

Chemistry–A European Journal

Supporting Information

Different Topologies of Hg(II)-Bispidine 1D Coordination Polymers: Dynamic Behavior in Solvent Adsorption and Exchange Processes

Martina Lippi, Andrea Murelli, Patrizia Rossi, Paola Paoli, and Massimo Cametti*

TABLE OF CONTENT:

Experimental details:

-Characterization of ligand **L3**--S2-S3

-Synthesis of CPs SCs--S4

-SC-XRD and In-silico analysis:--S5

-Crystallographic Tables: **Table S1** and **S2**---S6-S7

- **Table S3** Relevant distances and angles related to Hg(II) coordination--S8-S9

-Synthesis of CP powders--S10

- **Table S4** Reaction time of all synthesized microcrystalline CPs --S10

-Figure S6. Superimposition of the bispidine ligand **L3**--S11

-Figure S7. Ball&stick view of adjacent zig-zag 1D-CPs in **3·MeOH**^{SC}, **3·EtOH**^{SC}, **3·ClBz**^{SC}-------------------------S12

-Figure S8. View of the **c**rystal packings of **3·MeOH**^{SC}, **3·EtOH**^{SC}, **3·ClBz**^{SC}---S13

-Figures S9a-c Inter-array contacts in **3·MeOH**^{SC}, **3·EtOH**^{SC} and **3·ClBz**^{SC} ---S14

-Figures 10a-d. Hirschfeld surface plots for L3 in **3·MeOH**^{SC}, **3·EtOH**^{SC} and **3·ClBz**^{SC}-----------------------------S15

-Figure S11. Experimental PXRD of **3·ClBz**$^{Pwd-II}$--S16

-Figure S12. P-XRD: Comparison between zig-zag CPs---S17

-Figure S13 ^{1}H-NMR spectra of CPs before and after thermal treatment--------------------------------------S19-S20

-Figure S14 P-XRD patterns comparisons concerning **3·ClBz-polycatenane**^{Pwd}, **3·1,2-DCB**$^{pwd-I}$ and **3·1,2-DCB**$^{pwd-II}$ and **3·ClBz**$^{pwd-I}$. ---S21

-Figure S15 Views of the void space in **3·ClBz-polycatenane**^{SC} and discussion about solvents----------------S22

-Figure S16 P-XRD of **3·ClBz-polycatenane**^{Pwd} after thermal treatment at 80 °C and 120 °C -------------------S23

Figure S17 ^{1}H-NMR spectra **3·ClBz-polycatenane**^{Pwd} treated at 80°C and 120°C. --------------------------------S24

References--S25

Characterization of Ligand **L3**

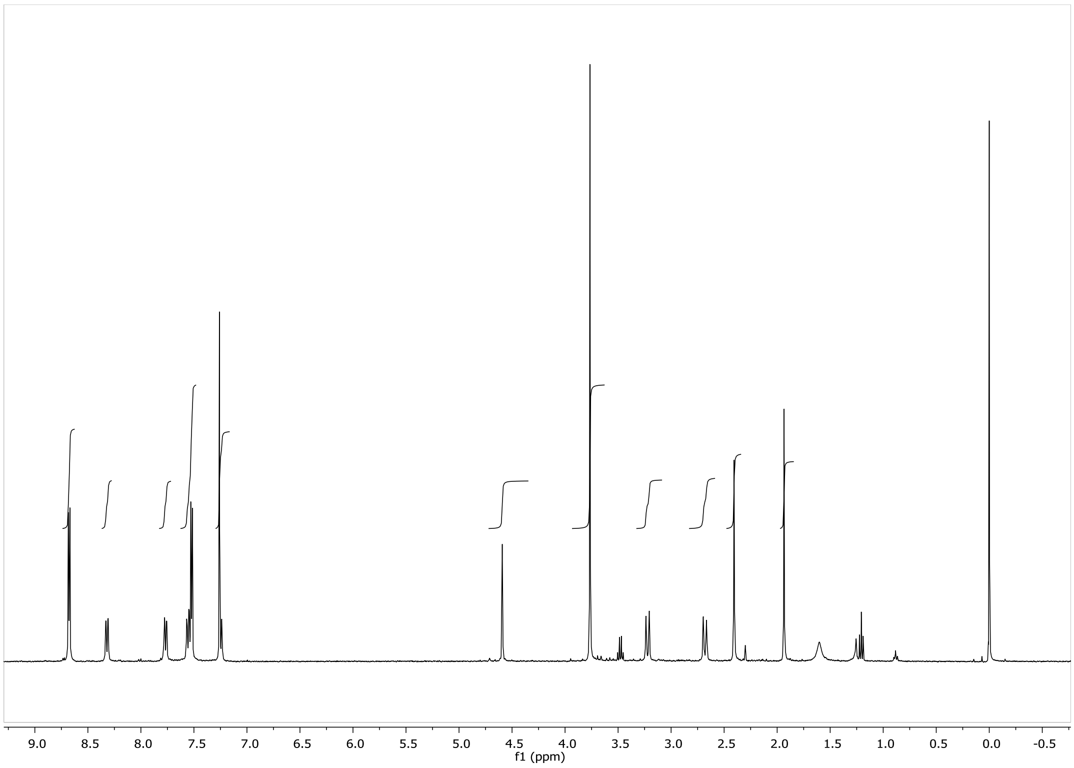

Figure S1. ^{1}H-NMR spectrum of **L3** ligand in DMSO-d_6.

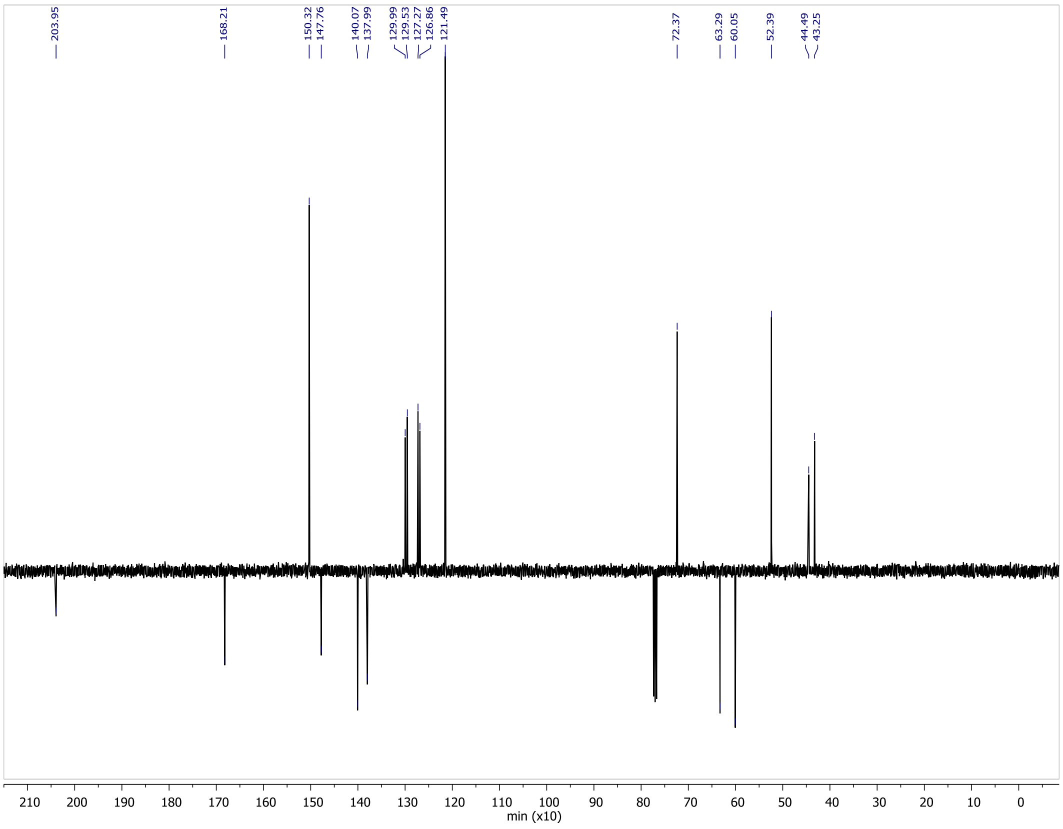

Figure S2. ^{13}C-NMR spectrum of **L3** ligand in DMSO-d_6.

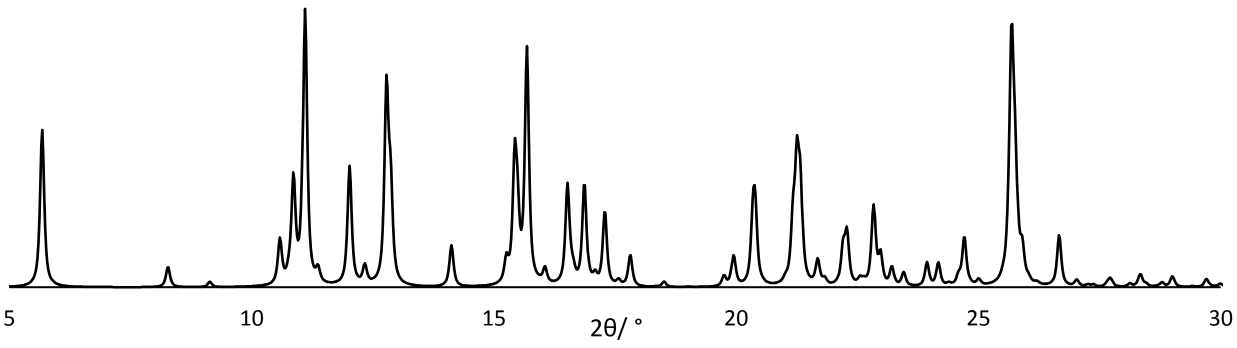

Figure S3. Experimental P-XRD pattern of bispidine derivative ligand **L3**.

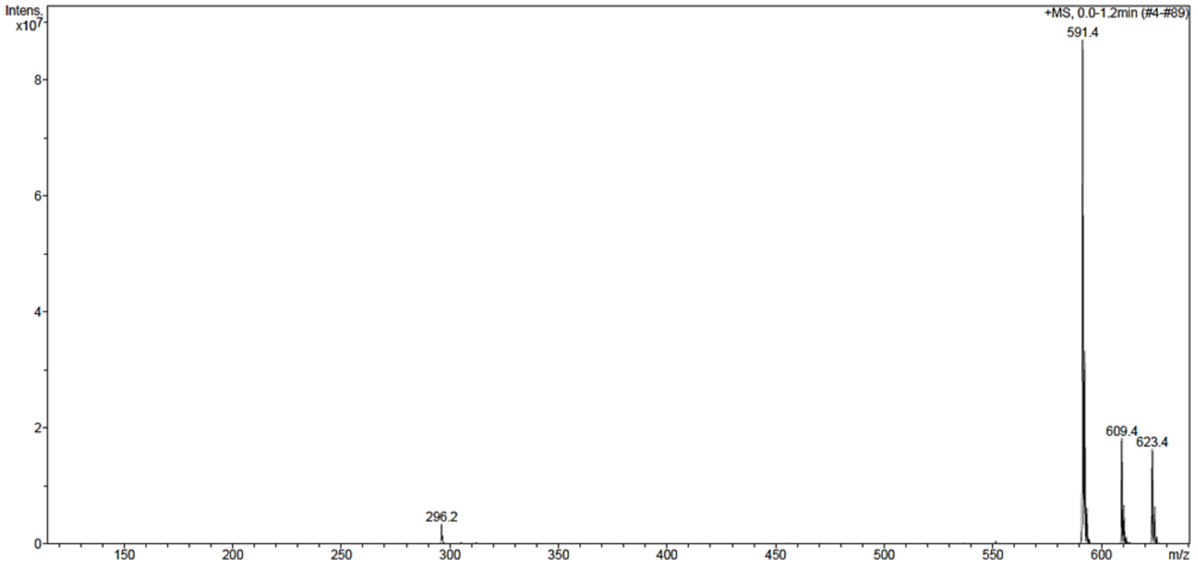

Figure S4. ESI-Mass spectrum of ligand **L3**.

Synthesis of CPs SCs:

Single Crystals of CPs made with ligand **L3** and $HgCl_2$ were obtained through a slow crystallization process with a wide range of solvents using the three-layer method shown in Figure S5. Good quality ones were obtained for **3·MeOH**^{SC}, **3·ClBz**^{SC}, **3·polycatenane**^{SC}, **3·EtOH**^{SC}, and **3·1,3CB**^{SC}. All of them were later submitted to SC-XRD analysis to characterize their structures. The implemented technique consists in the formation of 3 layers, each containing one of the compounds necessary for the construction of the CP in a vial or flask.

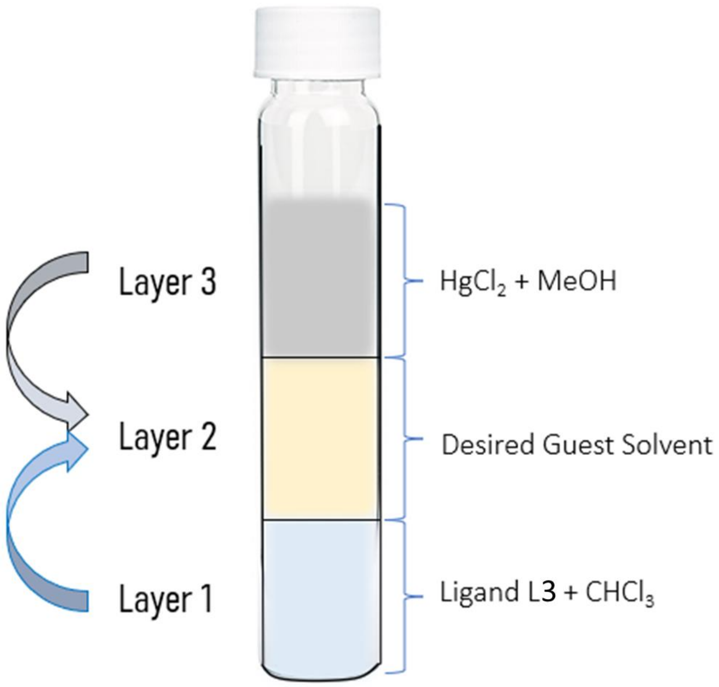

Figure S5. Schematic representation of the three-layer method: diffusion steps to allow the self-assembly of CPs.

Layer 1 consists of a 1 mL solution of chloroform and 25 mg of **L3** (0.043 mmol), while the intermediate layer is made out by 3 mL of the desired solvent, slowly introduced to avoid mixing with the first layer. The third layer is a solution of 1 mL of MeOH with 1 eq of $HgCl_2$ dissolved inside. The last layer must be introduced with extreme cure to avoid, again, the mixing of the 3 layers. After the layers are set, the vial is closed. If no single crystal is obtained in the first three days, a small hole in made to enhance the self-assembly process. After few days or weeks later, small, white crystals are formed.

SC_XRD characterization:

Intensity data from single crystal of **3·MeOH^{SC}**, **3·EtOH^{SC}**, **3·ClBz^{SC}**, **3·ClBz-polycatenane^{SC}** and **3·1,3-DCB^{SC}** were collected at 100K by using a Bruker APEX-II CCD equipped with a double X-ray source (Cu-K_{α} radiation was used for **3·MeOH^{SC}**, **3·ClBz^{SC}**, **3·ClBz-polycatenane^{SC}** and **3·1,3-DCB^{SC}**; Mo-K_{α} radiation was used for **3·EtOH^{SC}**). Data collections were performed with the program Bruker APEX2.[i] Data reductions were carried out with the program Bruker SAINT.[1ii] Finally, absorption corrections were performed with the program SADABS-2016/2.[iii]

Structures were solved by using the SIR-2004 package[iv] and subsequently refined on the F^2 values by the full-matrix least-squares program SHELXL-2013.[v]

Geometrical calculations were performed by PARST97,[vi] and molecular plots were produced by the program Mercury (2020.3.0)[vii]

In Table 1 crystal data and refinement parameters of the investigated structures are reported.

Except for the solvent molecules, all the non-hydrogen atoms of all the structures were refined by using anisotropic thermal parameters, while the hydrogen ones were set in calculated position and refined in agreement with the carbon atom to which they are bound.

In all the structures, most of the solvent molecules are affected by disorder which was handled using different strategies (*vide infra*).

The non-hydrogen atoms of the solvent molecules were isotropically refined and in some cases the hydrogen atoms were not introduced in the refinement. In particular:

a) **3·MeOH^{SC}**: in the asymmetric unit two **3·$HgCl_2$** units are present. The disorder affecting the methanol molecules was elucidated to 2.5 disordered molecules with partial occupancy factor over several sites. More in detail, 8 models were used to model the solvent molecules: 3 with occupancy factors 0.5, 0.35, 0.25, respectively; 3 with occupancy factor 0.2; 2 having occupancy factor 0.4.

b) **3·EtOH^{SC}** and **3·ClBz^{SC}** in the crystal structure **3·$HgCl_2$** crystallizes with half solvent molecule in the asymmetric unit. The ethanol molecule is disordered over two sites with equal occupancy factor (0.25). Two different orientations have been found for the chlorobenzene molecule, which are related by an inversion center (3 carbon atoms, 1 chlorine atom and 2 hydrogen atoms are in the asymmetric unit; the occupancy factor of the chlorine atom was set to 0.5).

c) **3·1,3-DCB^{SC}**: the Hg atom is located on a symmetry centre. As for the solvent molecule, the asymmetric unit contains one 1,3-DCB molecule at full occupancy and one with half occupancy factor. The latter being affected also by orientational disorder.

d) **3·ClBz-polycatenane^{SC}**: the Hg atom is located on a twofold symmetry axis. The asymmetric unit is completed by 1 chlorobenzene molecule affected by disorder (modelled over two sites with occupancy factor of 0.5) and an additional molecule completed by a 2-fold axis having occupancy factor 0.5 (two carbon atoms and the chlorine atom laying on the twofold axis, occupancy factor 0.25, the remaining carbon atoms with occupancy factor 0.5). The first chlorobenzene is orientationally disordered, the second one is affected by positional disorder.

In-silico analysis. Crystal packings were analyzed with Mercury.[vi] Crystal-Explorer17[viii] was used to compute the Hirshfeld surfaces (HS) in order to check the intermolecular interactions which hold together the 1D-CPs.

Table 1. Crystal data and refinement parameters of **3·MeOH**^{SC}, **3·EtOH**^{SC}, **3·ClBz**^{SC}.

	**3·MeOH**^{SC}	**3·EtOH**^{SC}	**3·ClBz**^{SC}
Empirical formula	[**L3·HgCl$_2$**]·1.25MeOH	[**L3·HgCl$_2$**]·0.5EtOH	[**L3·HgCl$_2$**]·0.5ClBz
Formula weight	1798.36	883.30	917.92
Temperature (K)	100	100	100
Wavelength (Å)	1.54178	0.71073	1.54178
Crystal system, space group	Triclinic, P-1	Monoclinic, P2$_1$/n	Monoclinic, P2$_1$/n
Unit cell dimensions (Å, °)	a = 18.7353(7), α = 90.105(1)<br>b =10.4719(4), β =110.194(1)<br>c = 19.4994(8), γ = 92.542(2)	a = 18.758(1)<br>b = 10.3146(7), β = 111.956(2)<br>c = 19.524(1)	a = 18.5590(5)<br>b = 10.5587(3), β = 108.620(1)<br>c = 19.5215(5)
Volume (Å^3)	3586.4(2)	3503.5(4)	3625.2(2)
Z, D_c (g/cm^3)	2, 1.665	4, 1.675	4, 1.682
μ (mm^{-1})	9.498	4.595	9.716
F(000)	1782	1752	1818
θ range (°)	2.415 – 72.901	2.249 – 24.763	3.983 – 72.301
Reflections collected / unique	93751 / 14124	34118 / 5987	60286 / 7141
Data / parameters	14124 / 911	5987 / 445	7141 / 460
Goodness-of-fit on F^2	1.069	1.047	1.087
Final R indices [I>2σ(I)]	0.0417 / 0.1069	0.0448 / 0.1044	0.0303 / 0.0763
R indices (all data	0.0435 / 0.1101	0.0691 / 0.1182	0.0322 / 0.0777

Table 2. Crystal data and refinement parameters of **3·ClBz-polycatenane**^{SC} and **3·1,3-DCB**^{SC}.

	**3·1,3-DCB**^{SC}	**3·ClBz-polycatenane**^{SC}
Empirical formula	[**L3·Hg$_{0.5}$Cl**]·1.5(1,3-DCB)	[**L3·Hg$_{0.5}$Cl**]·1.5ClBz
Formula weight	946.89	866.08
Temperature (K)	100	100
Wavelength (Å)	1.54178	1.54178
Crystal system, space group	Triclinic, P-1	Orthorhombic, Pccn
Unit cell dimensions (Å, °)	a = 9.9031(4), α = 103.794(2)<br>b =12.0494(5), β =99.989(2)<br>c = 18.5501(8), γ = 95.623(2)	a = 23.408(1)<br>b = 17.4187(7)<br>c = 19.777(1)
Volume (Å^3)	2094.5(2)	8064.0(7)
Z, D_c (g/cm^3)	2, 1.501	8, 1.427
μ (mm^{-1})	6.164	5.307
F(000)	960	3524
θ range (°)	2.505 – 68.745	5.072 – 89.589
Reflections collected / unique	50898 / 7719	60716 / 9171
Data / parameters	7719 / 516	9171 / 481
Goodness-of-fit on F^2	1.069	1.065
Final R indices [I>2σ(I)]	0.0474 / 0.1212	0.0518 / 0.1401
R indices (all data	0.0464 / 0.1235	0.0602 / 0.1470

Table S3. Selected distances and angles in **3·MeOH**^{SC}, **3·EtOH**^{SC}, **3·ClBz**^{SC}, **3·1,3-DCB**^{SC} and **3·ClBz-polycatenane**^{SC}

**Hg-X (Å)**	**3·MeOH**	**3·EtOH**	**3·ClBz**
Hg1-Cl1	2.348(1)	2.362(2)	2.378(1)
Hg1-Cl2	2.358(1)	2.352(2)	2.364(1)
Hg1-N1	2.424(1)	2.401(7)	2.373(1)
Hg1-N3^{1}	2.407(1)	2.424(7)	2.379(1)
Hg2-Cl3	2.362(1)		
Hg2-Cl4	2.384(1)		
Hg2-N5	2.360(1)		
Hg2-N7^{1}	2.357(1)		
**X-Hg-Y (°)**			
Cl1-Hg1-Cl2	150.6(1)	150.15(9)	143.5(1)
Cl1-Hg1-N1	95.6(2)	100.2(2)	97.12(7)
Cl1-Hg1-N3^{1}	96.7(1)	102.2(2)	95.5(1)
Cl2-Hg1-N1	101.2(1)	97.4(2)	106.85(7)
Cl2-Hg1-N3^{1}	102.7(1)	97.2(2)	105.8(1)
N1-Hg1-N3^{1}	100.9(1)	102.6(2)	101.58(9)
Cl3-Hg2-Cl4	143.2(1)		
Cl3-Hg2-N5	100.8(1)		
Cl3-Hg2-N7^{1}	100.8(1)		
Cl4-Hg2-N5	104.7(1)		
Cl4-Hg2-N7^{1}	100.2(1)		
N5-Hg2-N7^{1}	100.3(1)		
**THC_{DA}/100***	0.02 / 0.17	0.02	0.17
**τ_4****	0.76 / 0.80	0.75	0.78

^{1} = x,y,z+1

**Hg-X (Å)**	**3·1,3-DCB**	**3·ClBz-polycatenane**
Hg1-Cl1	2.453(1)	2.417(1)
Hg1-N1	2.553(4)	2.608(4)
Hg1-N3^{2}	2.558(4)	2.649(4)
**X-Hg-Y (°)**		
Cl1-Hg1-Cl1^{3}	180	179.84(9)

Cl1-Hg1-N1	90.27(9)	89.37(9)
Cl1-Hg1-N1[3]	89.7(1)	90.76(8)
Cl1-Hg1-N3[2]	89.2(1)	89.4(1)
Cl1-Hg1-N3[4]	90.8(1)	87.24(9)
N1-Hg1-N1[3]	180	77.1(1)
N1-Hg1-N3[2]	94.1(1)	93.9(1)
N1-Hg1-N3[4]	85.90(9)	170.3(1)
N1[2]-Hg1-N3[4]	180	93.9(1)

3·1,3-DCB: [2] = x-1,y,z-1; [3] = -x,-y,-z; [4] = -x+1,-y,-z+1

3·ClBz-polycatenane: [2] = x,y,z+1; [3] = -x+1/2,-y+1/2,z; [4] = -x+1/2,-y+1/2,z+1

$* THC_{DA} = \left(1 - \frac{\sum_{n=1-6}|109.5-\theta_n|}{90}\right) * 100$; θ_n is the n of six bond angles. $THC_{DA}/100$ value for trigonal pyramid is 0.00 (it is 1 for a tetrahedral coordination).[ix]

$** \tau_4 = \frac{360-(\alpha+\beta)}{141}$; α and β are the two largest h angles in the four-coordinate species. τ_4 value for trigonal pyramid is 0.85 (it is 1 for a tetrahedral coordination).[ix]

Synthesis of CPs powder

Fast crystallization. CPs in form of microcrystalline powders were synthesized through a fast crystallization process which consist in dissolving the ligand **L3** (50 mg) in 3 mL of guest solvent and then adding to this first solution a second solution of $HgCl_2$ (0.5 equivalents) in MeOH. The reaction mixtures were left under stirring at room temperature for a period of 30 minutes up to 2 hours. White powders were finally obtained and immediately collected by filtration.

Mechanochemical synthesis. In the case of crystallization in presence of chlorobenzene the synthesis by mechanochemical process was also performed as following: 1 Eq of ligand **L3** was mixed with 0.5 equivalents of $HgCl_2$ in a mortar and grinded for a couple of minutes to obtain a fine, properly mixed, powder. A few drops of ClBz were then added to it and grinding was resumed for 5-10 minutes, moving the product back to the center of the mortar from time to time to promote the synthesis process. At the end of the synthesis, the resulting white powder was left in open air to dry, collected and analyzed.

Table S4. Reaction time of all synthesized microcrystalline CPs.

CP	Synthetic method	Reaction time
**3·EtOH^{Pwd}**	Fast crystallization	20-30 min
**3·MeOH^{Pwd}**	Fast crystallization	20-30 min
**3·ClBz$^{Pwd\text{-}I}$**	Fast crystallization	20-30 min
**3·ClBz$^{Pwd\text{-}II}$**	Fast crystallization	>2h
**3·MeCN^{Pwd}**	Fast crystallization	20 min – 72 h
**3·MeBz^{Pwd}**	Fast crystallization	20 min – 72 h
**3·1,2DCB$^{Pwd\text{-}I}$**	Fast crystallization	20 min – 2h
**3·1,3DCB$^{Pwd\text{-}II}$**	Fast crystallization	20 min – 2h
**3·ClBz-polycatenane^{Pwd}**	Mechanochemical synthesis	15 min

X-ray Powder Diffraction

All the X-ray powder diffraction experiments were carried out using a Bruker D2-Phaser diffractometer equipped with Cu radiation (λ = 1.54184 Å) using Bragg-Brentano geometry. The experiments were performed at room temperature.

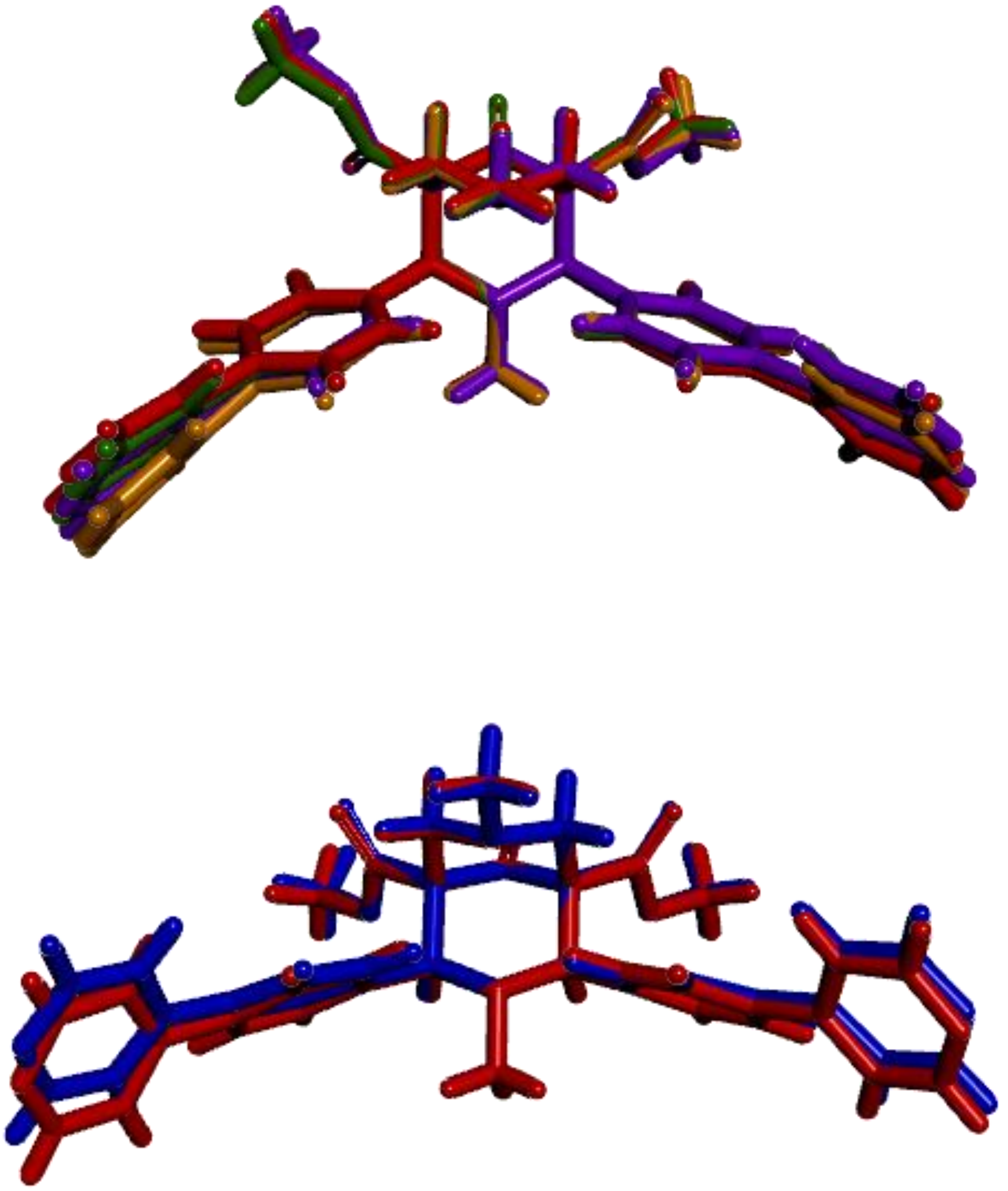

Figure S6. Superimposition of the bispidine ligand **L3**: top = **3·MeOH**^{SC} (A coordination complex, red), **3·EtOH**^{SC} (green), **3·ClBz-polycatenane**^{SC} (purple) and **3·1,3-DCB**^{SC} (orange); bottom = **3·MeOH**^{SC} (B coordination complex, red) and **3·ClBz**^{SC} (blue). Ligands were superimposed with respect to the carbonyl carbon atom and the two N-CH_3 atoms.

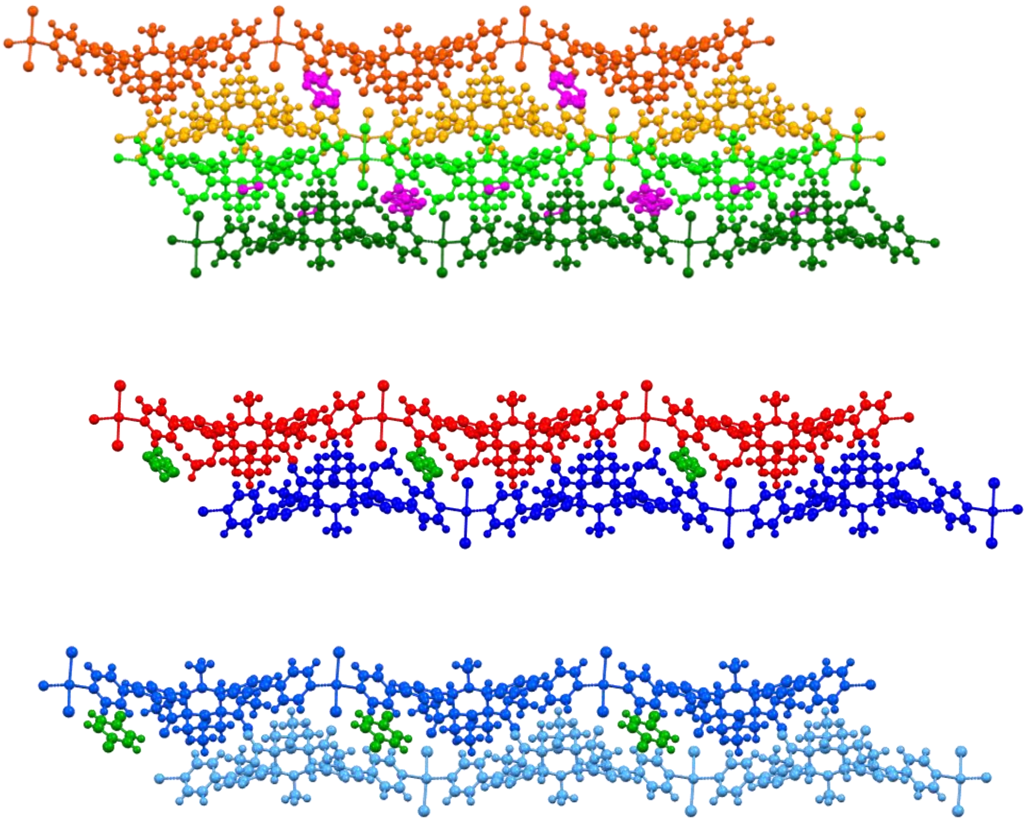

Figure S7. Ball&stick view of Adjacent zig-zag 1D-CP with solvent molecules in fuchsia and green colors for a) **3·MeOH**^{SC} extending along the c-axis direction ("*anti*" chains: green tones; "*syn*" chains orange tones); b) **3·EtOH**^{SC} extending along the c-axis direction and c) **3·ClBz**^{SC} extending along the c-axis direction.

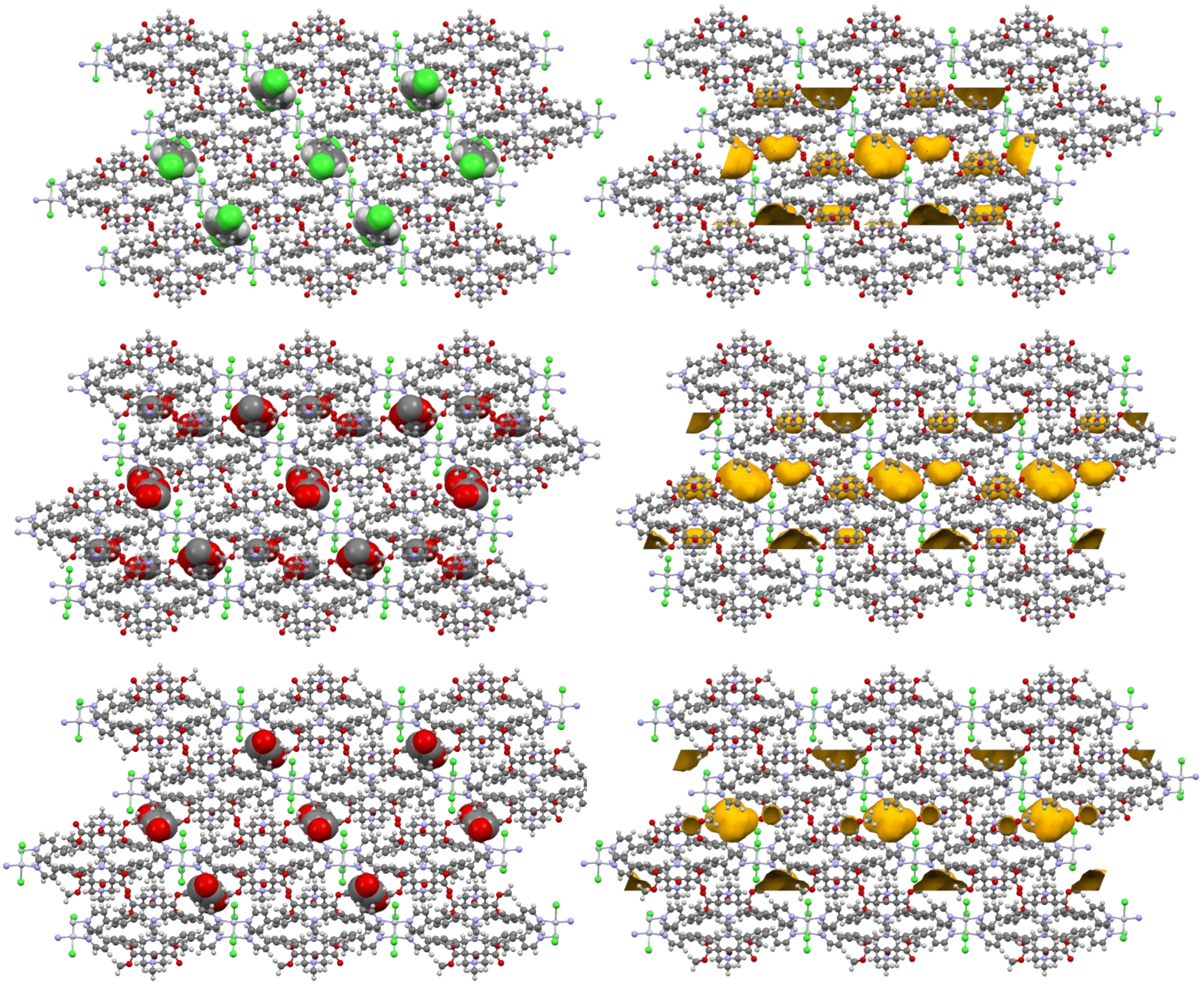

Figure S8. Crystal packings of **3·MeOH^{SC}** (top), **3·EtOH^{SC}** and **3·ClBz^{SC}** (bottom) view along the b axis direction. Disordered solvent molecules are displayed as spacefill (left); resulting voids in the unit cell from their in-silico removal are displayed in yellow (right).

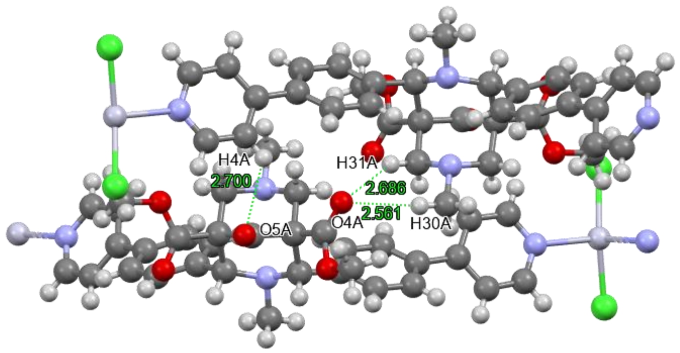

a

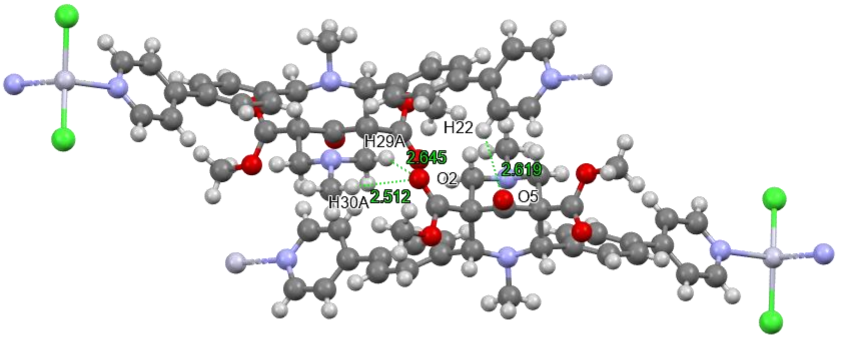

b

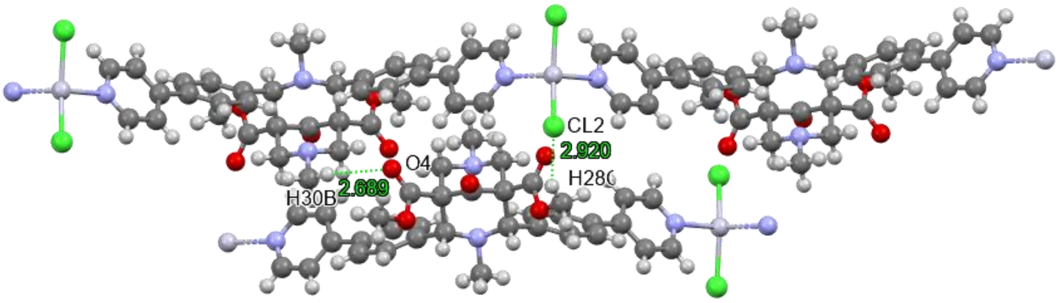

c

Figure S9. Inter-array contacts (O…H and Cl…H distances less than the sum of the corresponding VdW radii were considered)[x]: in **3·MeOH**^{SC} (a), **3·EtOH**^{SC} (b) and **3·ClBz**^{SC} (c).

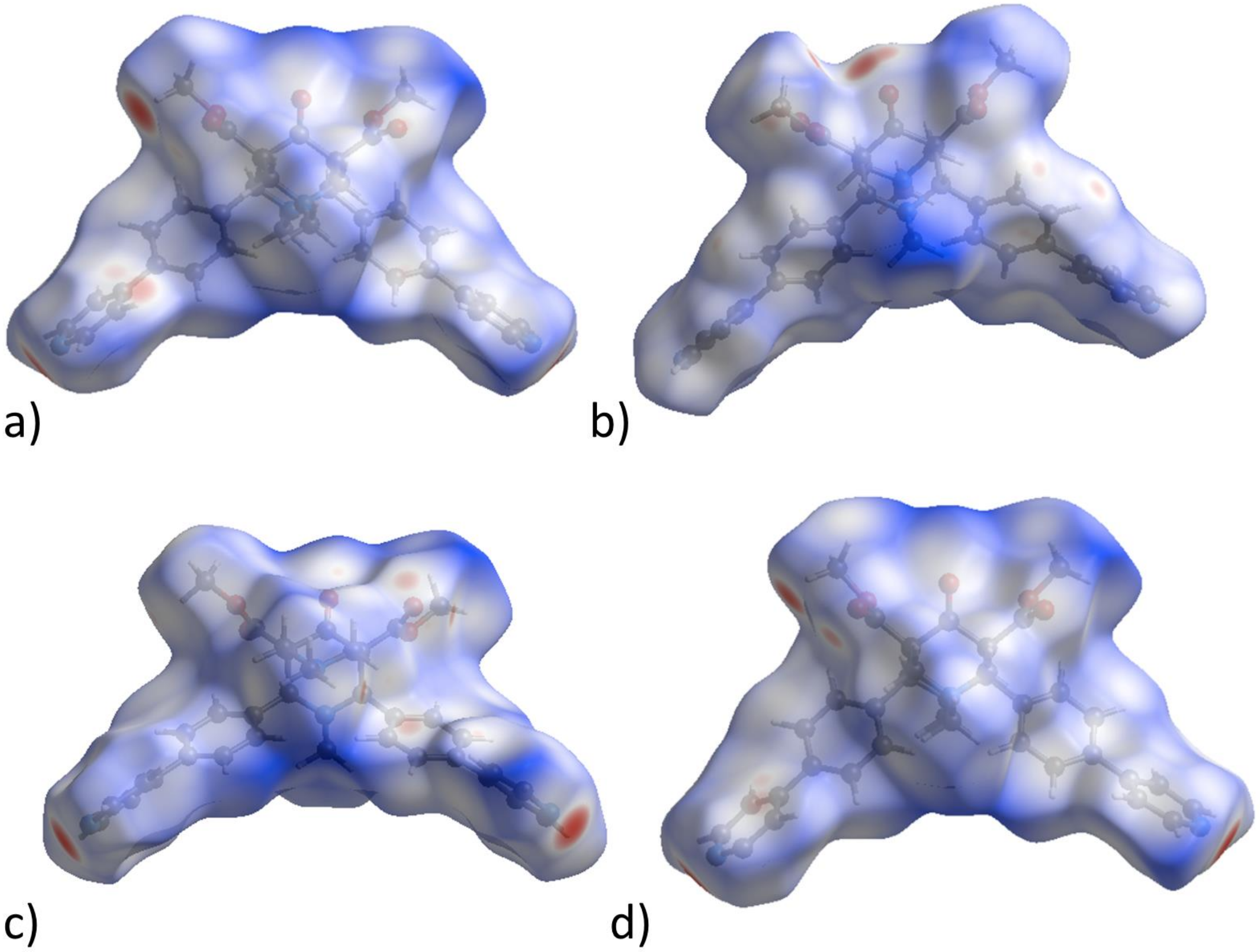

Figure S10. a) Hirshfeld surface of the *syn*-bispidine ligand in **3·MeOH**^{SC} (the red spot on the top left is due the intermolecular contact with the methanol molecule); b) Hirshfeld surface of the *anti*-bispidine ligand in **3·MeOH**^{SC} (the red spot on the top center are due the intermolecular contacts with the methanol molecule). c) Hirshfeld surface of the *anti*-bispidine ligand in **3·EtOH**^{SC}; d) Hirshfeld surface of the bispidine ligand in **3·ClBz**$^{SC\text{-}I}$ (the red spot on the top left is due the intermolecular contacts with the chlorobenzene molecule).

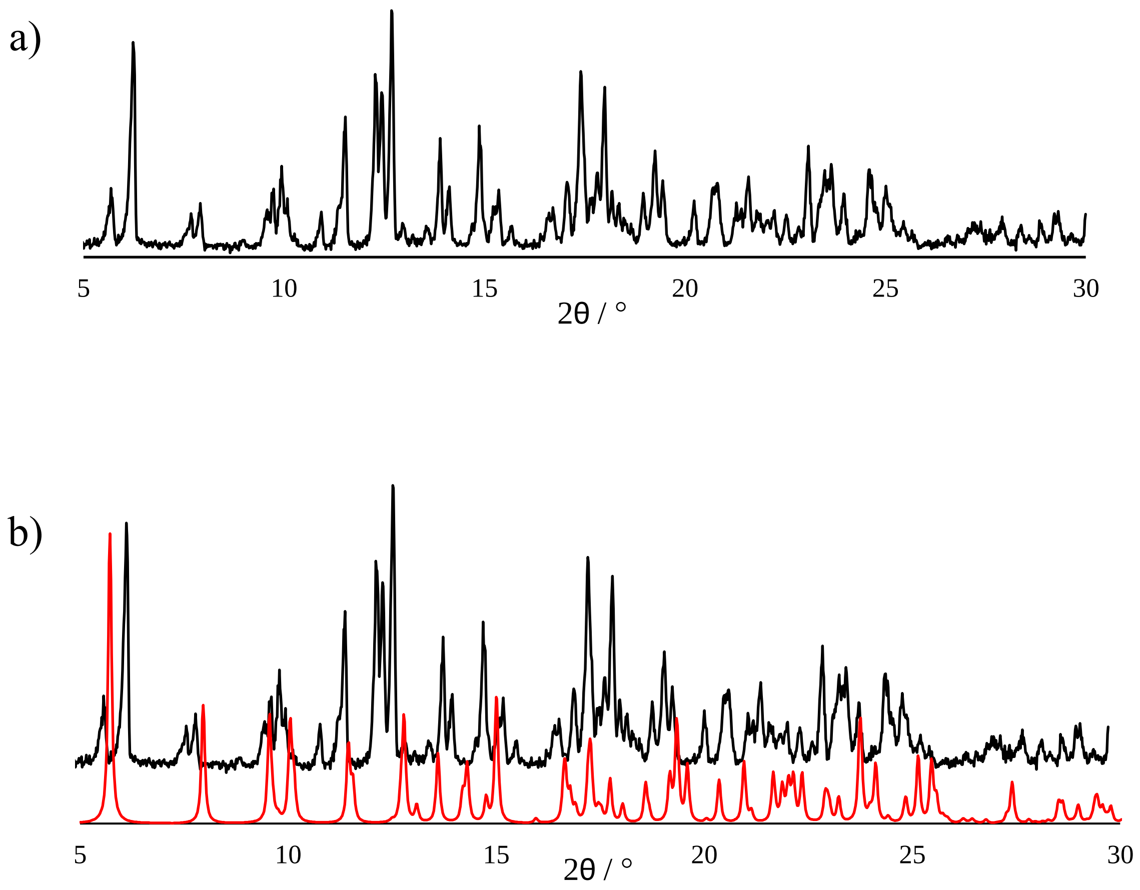

Figure S11. a) Experimental PXRD of **3·ClBz**$^{\mathbf{Pwd\text{-}II}}$ and b) the overlapping between a) and the simulated PXRD of **3·ClBz**$^{\mathbf{SC}}$.

a)

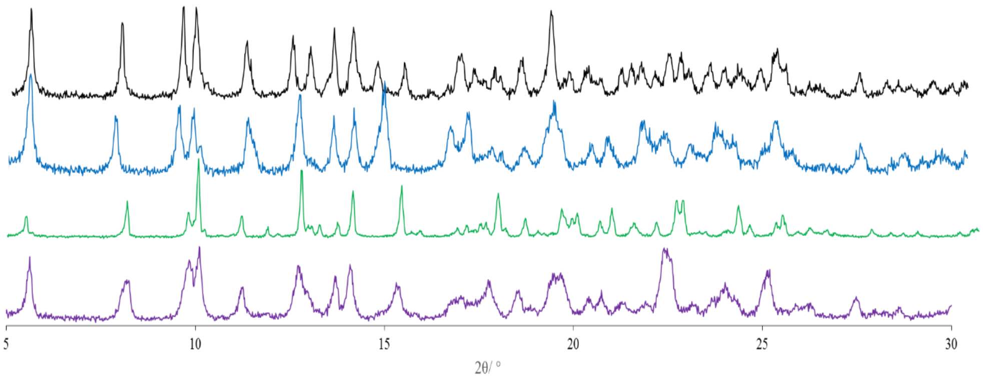

b)

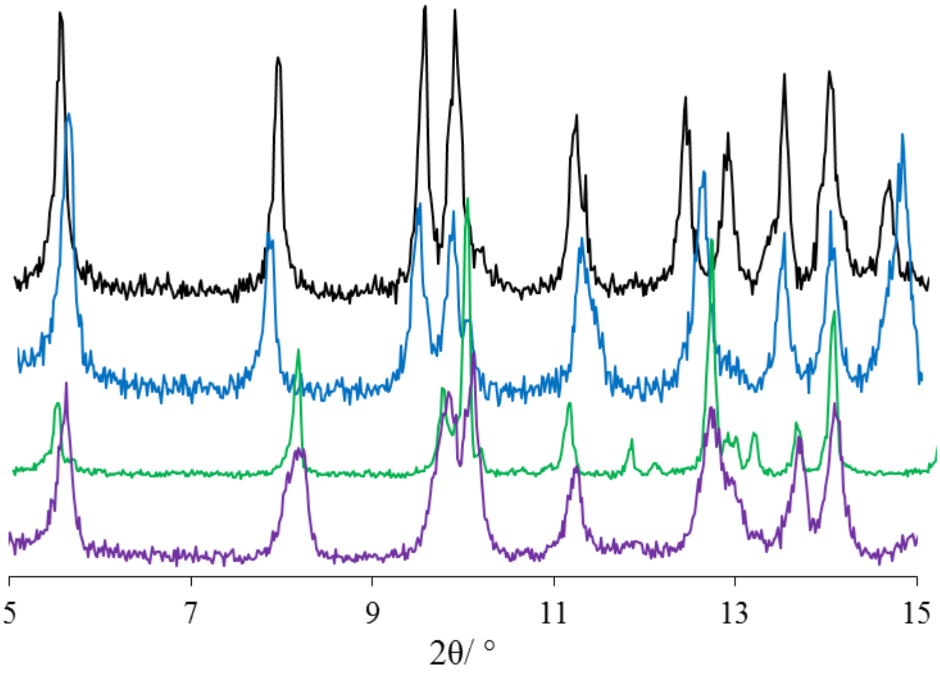

Figure S12. a) Overlapping between **3·ClBz^{Pwd-I}** (light blue), **3·MeOH^{Pwd}** (black), **3·EtOH^{Pwd}** (green), **3·MeCN^{Pwd}** (purple). b) Magnification of powder patterns showed in a) displaying signals from 5 to 15 2θ / °.

Guest exchange experiments: Procedure

Solid/liquid guest exchange experiments were performed by dipping the microcrystalline powder phase (40-50 mg) in a given solvent (5 mL) for 18 hours. Then, the powder was filtered and analysed by P-XRD. Solid/vapour exchange experiments concern instead the exposure of the powder to the vapors of a solvent by keeping the sample in a closed chamber where a certain amount of liquid volatile solvent was present.

Initial Phase	Solvent	Phase obtained after exposure to Solvent vapors	Phase obtained after dipping in liquid Solvent
**3·MeCN**^{pwd}	Methanol	No Transformation	No Transformation
**3·MeCN**^{pwd}	Chlorobenzene	No Transformation	No Transformation
**3·MeOH**^{pwd}	Acetonitrile	No Transformation	No Transformation
**3·MeOH**^{pwd}	Chlorobenzene	No Transformation	No Transformation
**3·CB**$^{pwd\text{-}I}$	Methanol	No Transformation	No Transformation
**3·CB**$^{pwd\text{-}I}$	Acetonitrile	No Transformation	No Transformation

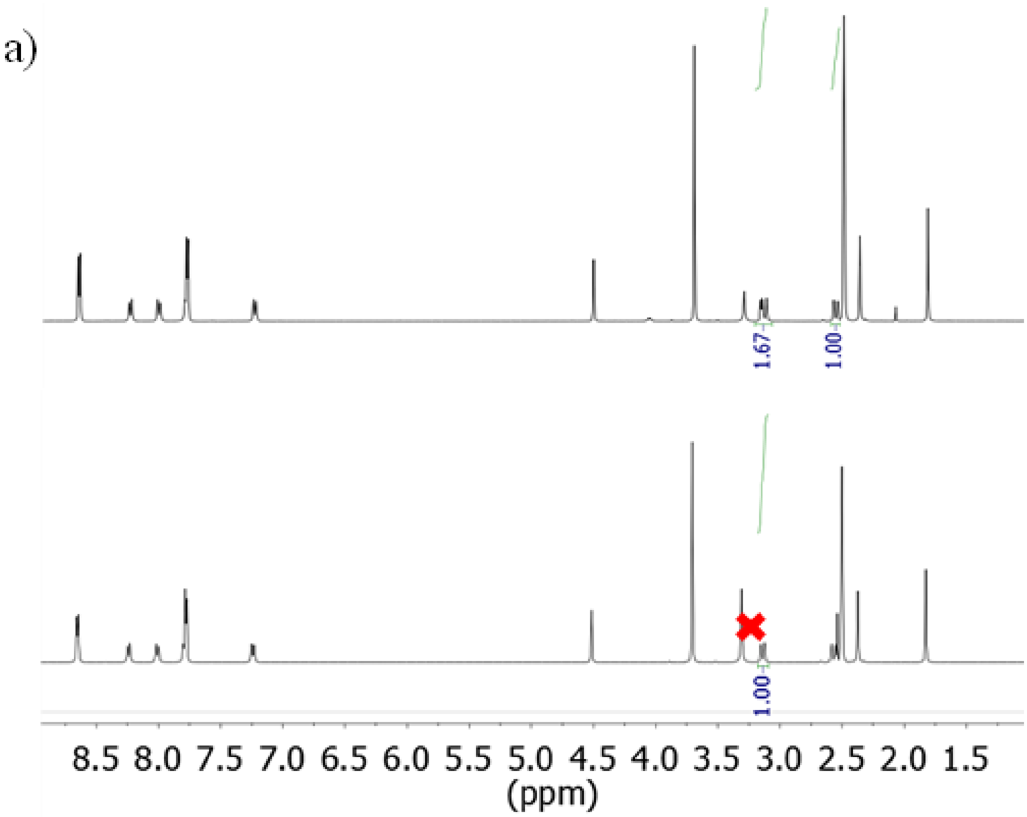
a)
1.67
1.00
1.00
8.5 8.0 7.5 7.0 6.5 6.0 5.5 5.0 4.5 4.0 3.5 3.0 2.5 2.0 1.5
(ppm)

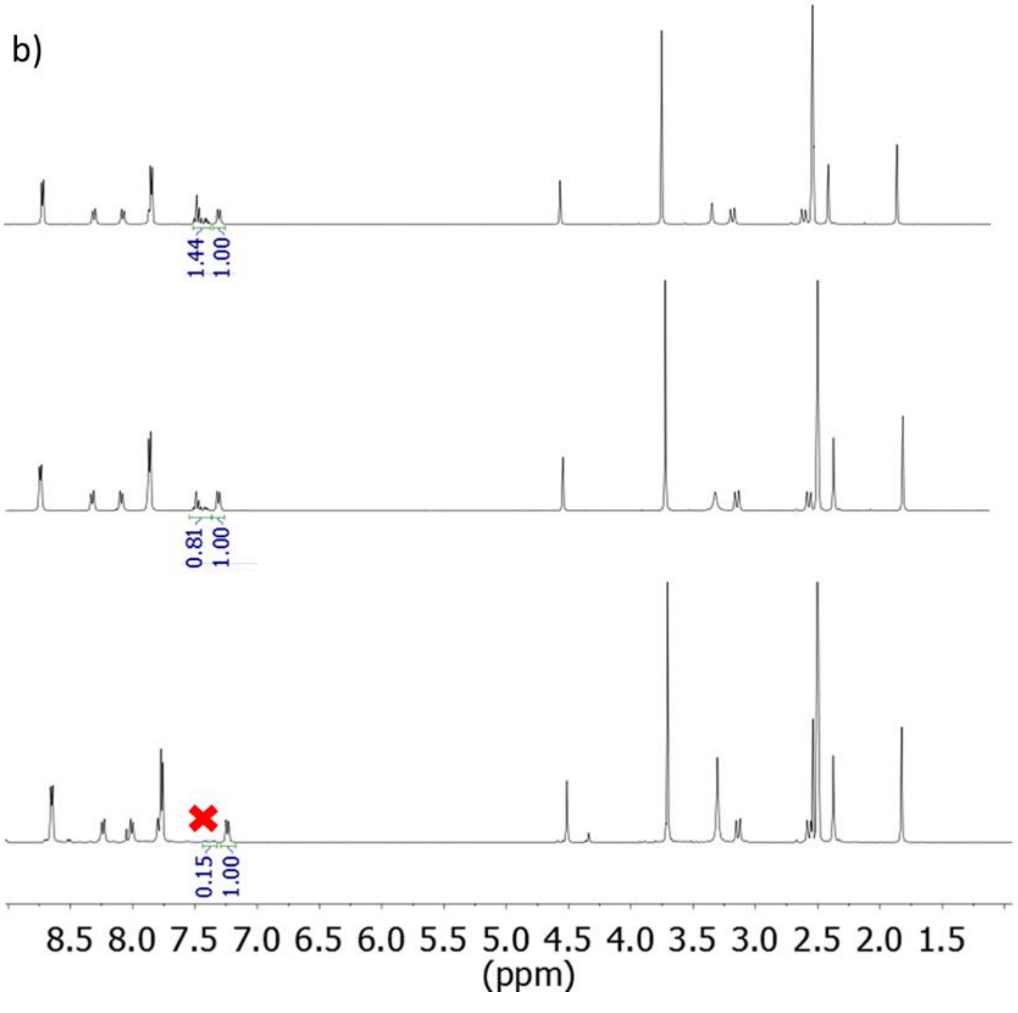
b)
1.44
1.00
0.81
1.00
0.15
1.00
8.5 8.0 7.5 7.0 6.5 6.0 5.5 5.0 4.5 4.0 3.5 3.0 2.5 2.0 1.5
(ppm)

c)

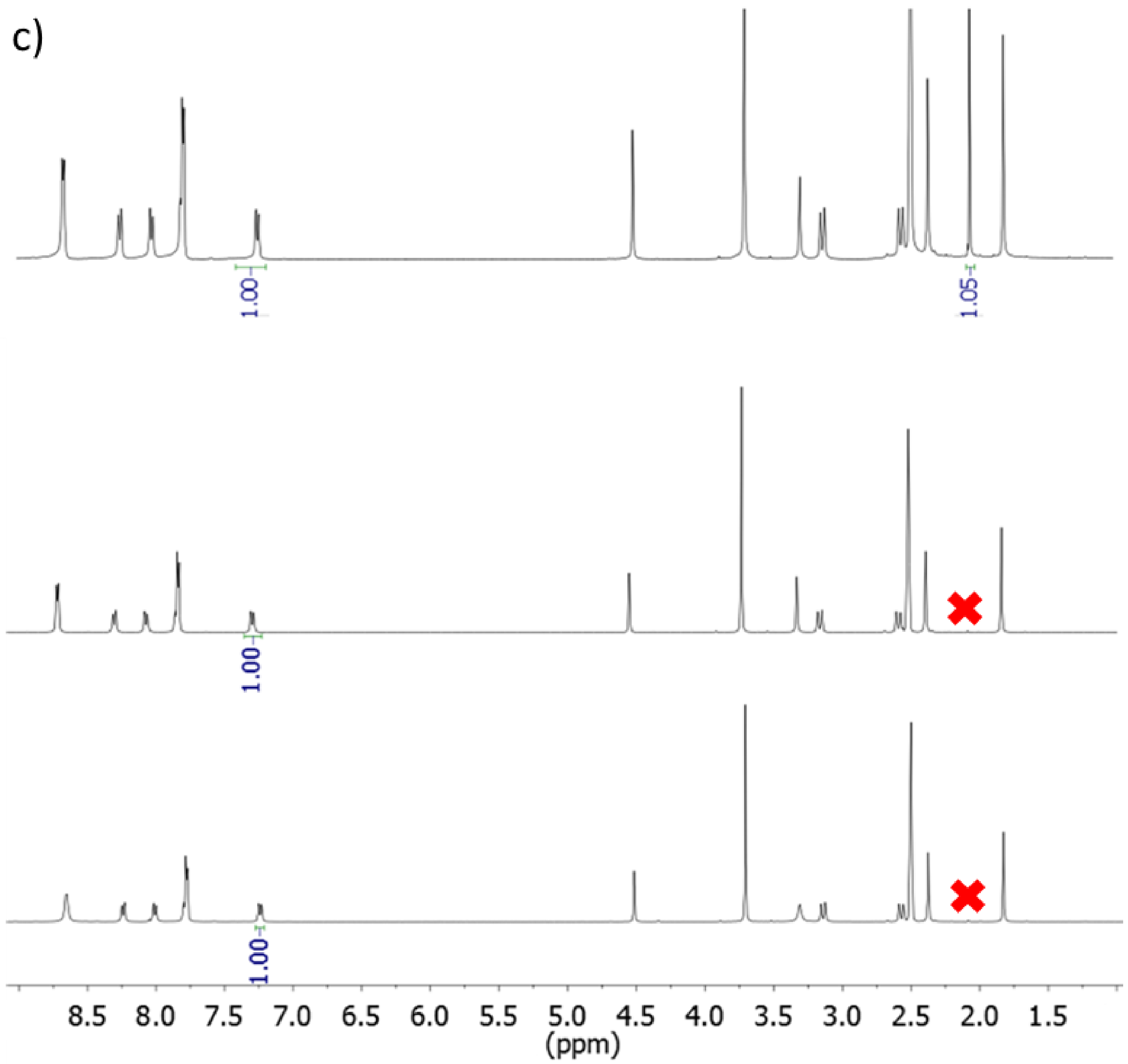

Figure S13. a) ^{1}H-NMR spectra of **3·MeOH^{Pwd}** (top) and **3·desolv^{Pwd}** (bottom) obtained after a thermal treatment at 80°C (18h) of **3·MeOH^{Pwd}**; b) ^{1}H NMR spectra of **3·ClBz$^{Pwd\text{-}I}$** (top) and **3·ClBz$^{Pwd\text{-}I}$** thermally treated at 80°C for 18h (middle) and 120°C for 18h (bottom); c) ^{1}H NMR spectra of **3·MeCN^{Pwd}** (top) and **3·MeCN^{Pwd}** thermally treated at 80°C for 18h (middle) and 120°C for 18 h (bottom).

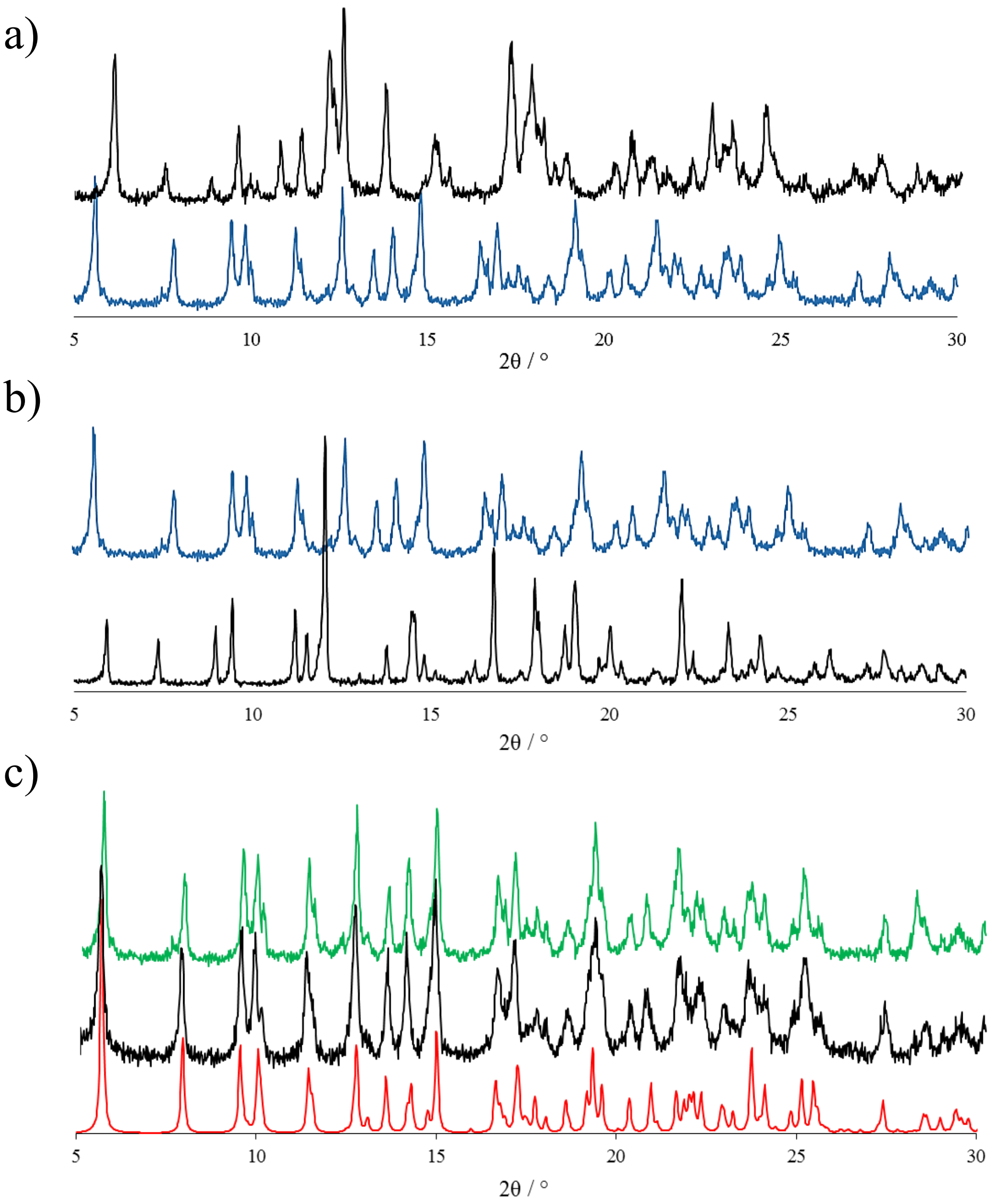

Figure S14. a) Overlapping between experimental P-XRD patterns of **3·ClBz-polycatenane**^{Pwd} (black) and the one obtained after dipping it in 1,2-dichlorobenzene for 18 hours (named **3·1,2-DCB**$^{\text{pwd-II}}$, blue); **b)** overlapping between **3·1,2-DCB**$^{\text{pwd-II}}$ (blue) and **3·1,2-DCB**$^{\text{pwd-I}}$ (black) obtained by instant synthesis; **c)** overlapping between P-XRD pattern obtained by dipping **3·1,2-DCB**$^{\text{pwd-II}}$ in chlorobenzene for 18 h (green), experimental P-XRD of **3·ClBz**$^{\text{pwd-I}}$ obtained by instant synthesis (black) and the simulated P-XRD pattern of **3·ClBz**^{sc} (red).

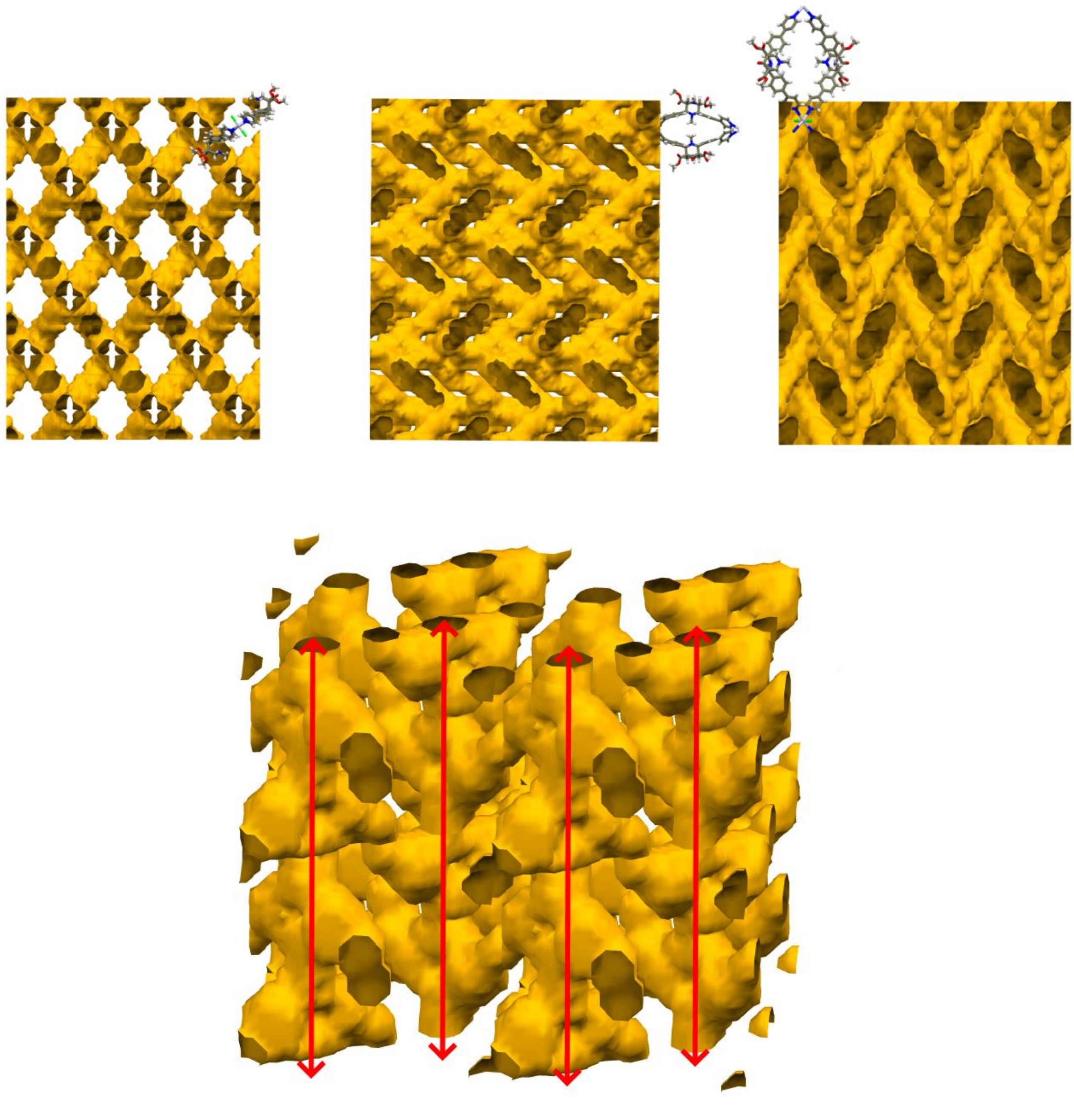

Figure S15. Views of the void space generated by virtual removal of the ClBz solvent molecules present in **3·ClBz-polycatenane^{SC}.** As to the included ClBz solvents, disorder is orientational (ClBz_O, which was taken into account with two models) and positional (ClBz_P, occupancy factor 0.50). ClBz molecules repeat in the inter chain space giving rise to the sequence ClBz_P-ClBz_O-ClBz_O-ClBz_P. The "dimer" ClBz_O-ClBz_O is hosted between two adjacent polycatenanes, while the ClBz_P molecules are squeezed between four ClBz_O solvent type molecules. The in-silico removal of the positionally disordered solvent molecules results in 7.9% of empty space and originates isolated voids repeating along the c-axis direction. The selective removal of the ClBz_O solvent type molecules frees 17.9% of space and results in a more complex picture of the empty volume both in terms of shape (elongated cocoons hosting two ClBz_O molecules) and distribution. The complete removal of the chlorobenzene molecules originate channels extending along the c-axis direction (due to the lack of ClBz_P molecules) interconnected to other channels due to the removal of the ClBz_O molecules.

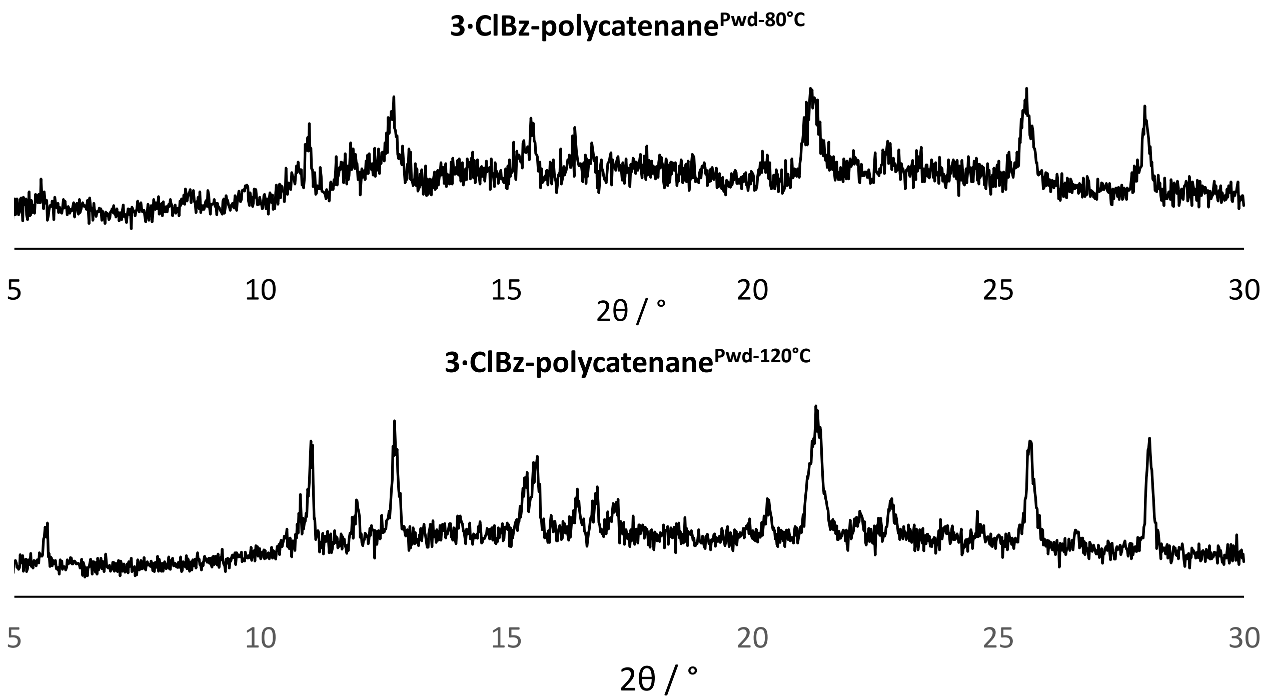

Figure S16. Experimental PXRD patterns of **3·ClBz-polycatenane**^{Pwd} after thermal treatment at 80 °C (above) and 120 °C (below).

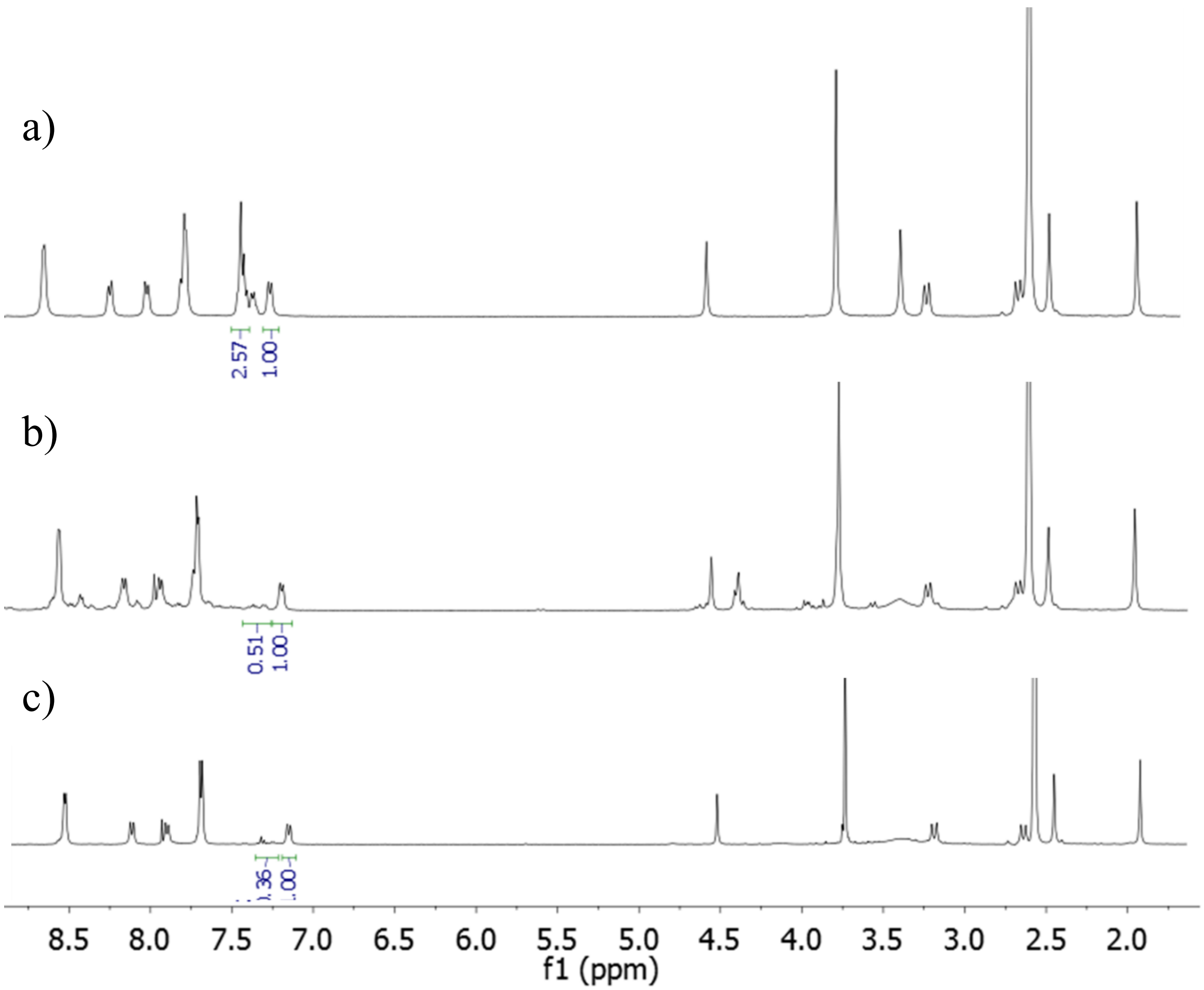

Figure S17. ^{1}H-NMR spectra of a) **3·ClBz-polycatenane^{Pwd}** as-synthesized and after thermal treatment at b) 80°C (18h) and c) 120°C (18h).

i Bruker (2012). Bruker *APEX2*. Bruker AXS Inc., Madison, Wisconsin, USA.

ii Bruker (2012). Bruker *SAINT*. Bruker AXS Inc., Madison, Wisconsin, USA.

iii Krause, L., Herbst-Irmer, R., Sheldrick G.M. & Stalke D. *J. Appl. Cryst.*, **2015**, 48,3-10.

iv Burla, M.C.; Caliandro, R.; Camalli, M.; Carrozzini, B.; Cascarano, G. L.; Da Caro, L.; Giacovazzo, C.; Polidori, G.; Spagna, R. J. Appl. Cryst., **2005**, *38*, 381-388.

v Gruene, T.; Hahn, H. W.M; Luebben, A. V.; Meilleur, F.; Sheldrick, G. M. J. Appl. Cryst., **2014**, *47*, 462-466.

vi Nardelli,M. *J. Appl. Cryst*, **1995**, *28*, 659.

vii Macrae, C. F.; Bruno, I. J.; Chisholm, J. A.; Edgington, P. R.; McCabe, P.; Pidcock, E.; Rodriguez-Monge, E.; Taylor, R.; van de Streek; J. Wood, P. A. *J. Appl. Crystallogr*. **2008**, *41*, 466-470.

viii Turner, M. J., McKinnon, J. J., Wolff, S. K., Grimwood, D. J., Spackman, P. R., Jayatilaka, D.; Spackman, M. A. Crystal Explorer17; University of Western Australia, 2017.

ix Lei Yang, Douglas R. Powell and Robert P. Houser, *Dalton Trans.*, **2007**, 955–964

x Bondi, A. *J. Phys. Chem*. **1964**, *68*, 441-461.
